# CFTR represses a PDX1 axis to govern pancreatic ductal cell fate

**DOI:** 10.1016/j.isci.2024.111393

**Published:** 2024-11-15

**Authors:** Pavana G. Rotti, Yaling Yi, Grace Gasser, Feng Yuan, Xingshen Sun, Idil Apak-Evans, Peipei Wu, Guangming Liu, Soon Choi, Rosie Reeves, Attilina E. Scioneaux, Yulong Zhang, Michael Winter, Bo Liang, Nathan Cunicelli, Aliye Uc, Andrew W. Norris, Lori Sussel, Kristen L. Wells, John F. Engelhardt

**Affiliations:** 1 Whitehead Institute, MIT, Cambridge, MA, USA; 2 Department of Anatomy and Cell Biology, Carver College of Medicine, University of Iowa, Iowa City, IA, USA; 3 Center for Gene Therapy, Carver College of Medicine, University of Iowa, Iowa City, IA, USA; 4 Stead Family Department of Pediatrics, Carver College of Medicine, Iowa City, IA, USA; 5 Barbara Davis Center for Childhood Diabetes, University of Colorado Anschutz, Medical Campus, Aurora, CO, USA; 6 Department of Anatomy and Cell Biology, Carver College of Medicine, University of Iowa, Iowa City, IA, USA

**Keywords:** Physiology, Molecular biology, Cell biology, Transcriptomics

## Abstract

Inflammation, acinar atrophy, and ductal hyperplasia drive pancreatic remodeling in newborn cystic fibrosis (CF) ferrets lacking a functional cystic fibrosis conductance regulator (CFTR) channel. These changes are associated with a transient phase of glucose intolerance that involves islet destruction and subsequent regeneration near hyperplastic ducts. The phenotypic changes in CF ductal epithelium and their impact on islet function are unknown. Using bulk RNA sequencing (RNA-seq), single-cell RNA sequencing (scRNA-seq), and assay for transposase-accessible chromatin using sequencing (ATAC-seq) on CF ferret models, we demonstrate that ductal CFTR protein constrains PDX1 expression by maintaining PTEN and GSK3β activation. In the absence of CFTR protein, centroacinar cells adopted a bipotent progenitor-like state associated with enhanced WNT/β-Catenin, transforming growth factor β (TGF-β), and AKT signaling. We show that the level of CFTR protein, not its channel function, regulates PDX1 expression. Thus, this study has discovered a cell-autonomous CFTR-dependent mechanism by which *CFTR* mutations that produced little to no protein could impact pancreatic exocrine/endocrine remodeling in people with CF.

## Highlights

• The lack of CFTR leads to activation of PDX1 in pancreatic ductal epithelium
• CFTR-KO duct epithelium has open chromatin at endocrine regulatory loci
• Centroacinar cell expansion is observed in CFTR-KO epithelium
• The absence of CFTR protein inhibits PTEN and activates WNT and PDX1 expression

## Graphical abstract

**Figure:**
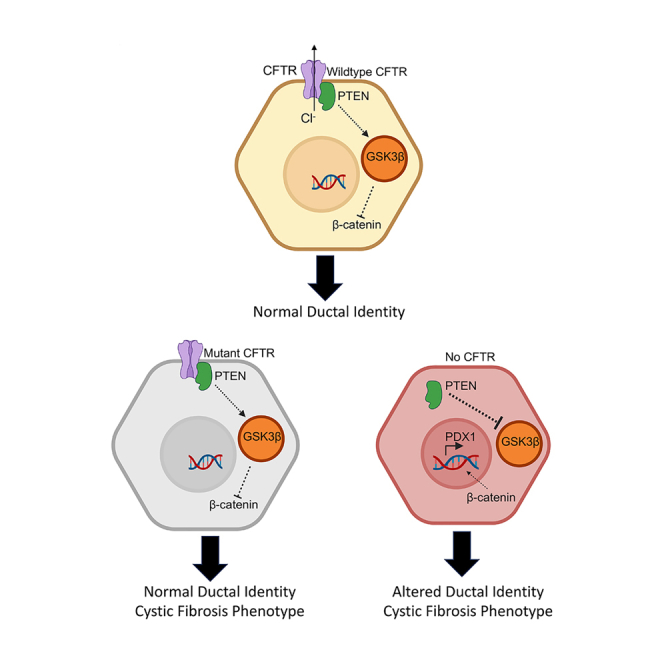

## Introduction

Cystic fibrosis (CF) is caused by mutations in the cystic fibrosis conductance regulator (CFTR), a chloride and bicarbonate channel that plays important roles in regulating the hydration and pH of epithelial secretions in the lung, pancreas, gallbladder, liver, and intestine.^1^ The pancreas is one of the earliest affected organs in CF, due to the lack of CFTR-mediated bicarbonate secretion into pancreatic ducts, which maintains an alkaline pH required to inhibit premature activation of pancreatic enzyme prior to exit into the intestine.^1^^,^^2^ In the absence of a functional CFTR channel, pancreatic acinar cell loss, severe inflammation, fibrosis, and adipogenesis alter the microenvironment of the pancreatic islets and ductal epithelium leading to pancreatic insufficiency and diabetes.^3^^,^^4^ At a histologic level, initial stages of CF pancreatic remodeling share similarities with developing pancreatic adenocarcinoma (PDAC), where hyperproliferative ducts form budding structures through a process thought to involve acinar to ductal metaplasia (ADM).^5^^,^^6^ However, little is known about the phenotypic alterations that occur in CF ductal cells and their impact on the function of islets within the pathologically altered pancreatic environment.^3^^,^^7^

CF pancreatic disease and its associated diabetes have been particularly difficult to study in mice lacking the *CFTR* gene. While certain genotypes of CF mice appear to exhibit some endocrine dysfunction and have reduced islet mass,^8^^,^^9^^,^^10^ they fail to develop major exocrine disease in the pancreas like humans.^7^ However, CF ferret models have proven useful to study pancreatitis and CF-related diabetes (CFRD) due to greater organ level conservation with human.^2^^,^^7^^,^^11^ We have previously categorized pancreatic tissue remodeling and associated abnormalities in glucose tolerance broadly into 4 phases using a *CFTR-KO* (CF) ferret model.^12^ A period of normoglycemia (phase I) is followed by a period of spontaneous glycemic instability at ∼1- to 2-months of age (phase II) with accompanied loss of endocrine hormone producing islets, severe fibrosis, and inflammation. This is followed by a transient recovery at ∼3-month of age (phase III) with continued tissue remodeling (adipogenesis) and temporary normalization of glucose tolerance during a period of islet resurgence.^12^ As CF ferrets age (phase IV), they then go on to develop the more classically studied CFRD. Similar to PDAC, CF pancreatic ducts proliferate and form budding structures that contain endocrine hormone-expressing cells during these transitions.^6^ Furthermore, similar phases of transient glucose intolerance followed by recovery are observed in young children with CF.^13^^,^^14^

Previous studies have implicated phenotypic alterations to *CFTR*-KO ferret ductal epithelium that can alter islet function through paracrine signaling of proinflammatory factors such as interleukin (IL)-6^15^ and trophic factors such as IGFBP7.^16^ Further support for altered exocrine to endocrine signaling in the *CFTR*-KO ferret pancreas comes from analyses of the pancreatic ductal secretome and whole cell proteome.^16^ These studies have implicated CF-associated alterations in transforming growth factor β (TGF-β), bone morphogenetic protein (BMP), phosphatase and tensin homolog (PTEN), AKT, wingeless-related integration site (WNT/β-catenin), and pancreatic duaodenal homeobox-1 (PDX1) signaling pathways and changes in the expression of proteins that directly bind with CFTR.^16^ Lastly, analysis of autocrine-paracrine circuits between exocrine/endocrine cell type, derived from 7 different human pancreatic islet single-cell RNA sequencing (scRNA-seq) datasets (7,603 cells), led to the discovery that BMP and WNT signaling is altered in the CF pancreas devoid of acinar cells.^17^ Despite the growing evidence for phenotypic changes in exocrine and endocrine cell types in the CF pancreas, the molecular mechanisms for these changes have remained elusive.

The PDX1 transcription factor is considered a master regulator of pancreatic development serving critical roles in the initiation of pancreas formation, the formation of multipotent progenitors in ductal epithelium, and the specification and maintenance of beta cells.^18^^,^^19^^,^^20^ Expression of both PDX1 and SOX9 in the duct epithelium facilitates an endocrine differentiation program via NGN3.^21^ However, ectopic expression of PDX1 in acinar cells has been shown to induce *trans*-differentiation to endocrine lineages.^22^ PDX1 also appears to play a critical role in the initiation of PDAC and progression during epithelial-to-mesenchymal transition (EMT).^23^ The mechanistic basis of aberrant PDX1 expression and its role in progression of pancreatic malignancies are still largely unknown. Integral to EMT in the progression of malignancies is the loss of apical-basolateral polarity maintained by apical anchoring proteins such as PTEN.^24^^,^^25^ In this context, PTEN has been shown to constrain centroacinar cell identity and loss of PTEN leads to the expansion of PDX1-expressing ductal cell, acinar cell loss, and PDAC.^26^

Here we characterized the transcriptomic and epigenomic landscape of wild-type (WT) and CF ferret pancreatic ductal epithelia to investigate whether mechanisms of ADM could explain genotypic changes previously observed in the ductal epithelial proteome and secretome.^16^ These studies discovered that high-level PDX1 expression in *CFTR*-knockout (KO) ductal epithelia likely originates from expansion of centroacinar cells with an altered epigenetic landscape that confers EMT-like features with differentially open chromatin at endocrine lineage associated transcription factor loci. Notably, these altered properties of CF pancreatic ductal epithelia were dependent on the loss of the CFTR protein, but not its channel function, and were driven by inhibition of the PTEN/GSK3β pathway leading to enhanced Wnt/β-catenin signaling. These findings suggest that CFTR residence on the apical membrane of ductal progenitors regulates their cell fate and may have implications for disease phenotypes in people with CF that harbor CFTR mutations that produce no or little protein.

## Results

### Pancreatic ductal epithelium activates PDX1 expression in *CFTR*-KO ferrets

**Figure 1:**
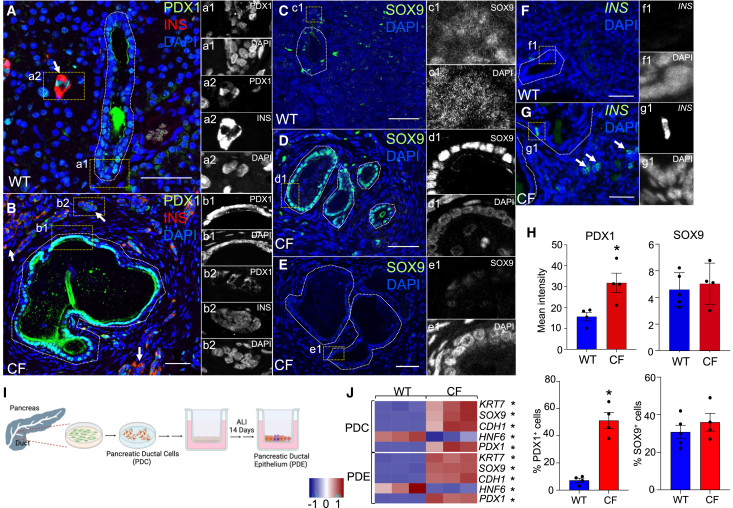
Aberrant gene expression in CF pancreatic ducts (A–E) WT and CF 2-month-old ferret pancreas immunostained for (A and B) PDX1 and INS or (C–E) SOX9. Insets are single-channel images of the regions marked by the dotted boxes. (F and G) Immunofluorescent images of 2-month-old WT and CF ferret pancreas stained for INS. Islets are identified by the expression of insulin and are marked by arrows. Ducts are identified by the presence of a lumen and marked by dotted lines. All images were acquired on a confocal microscope (Zeiss 880) at 20X magnification and processed for maximum intensity projection. Scale bars are 50 μm. (H) Mean intensity of nuclear PDX1 or SOX9 immunoreactivity within ductal cells and frequency of PDX1- or SOX9-positive cells from WT (*n* = 4 donors) and CF (*n* = 4 donors) 2-month-old pancreata. Four ducts were quantified from each donor and averaged. Data show the mean ± SEM. Nonparametric Mann-Whitney t test was used to evaluate significance (∗*p* < 0.05). (I) Schematic of *in vitro* approached used to establish ferret pancreatic duct epithelium (PDE) from passaged pancreatic ductal cells (PDCs). (J) Relative expression of pancreatic duct enriched genes in WT and CF PDC and PDE quantified using RT-qPCR. Nonparametric Mann-Whitney t test was used to evaluate significance (∗*p* < 0.05).

Given the shared features in pancreatic histopathology between CF and PDAC, we hypothesized that adaptive mechanisms that drive ADM may be similar. PDAC is frequently associated with the aberrant expression of pancreatic development genes PDX1^5^^,^^23^ and SOX9,^21^^,^^27^^,^^28^ which are required transcription factor for pancreas formation and PDAC progression. To this end, we evaluated expression of PDX1 and SOX9 in the *CFTR*-KO ferret pancreas at the stage of disease (∼2-month-old) associated with peak inflammation, fibrosis, and glycemic instability.^12^^,^^13^ Consistent with ADM-like features, expression of these master regulators of the pancreatic development program was elevated in a subset of *CFTR*-KO (CF) ductal epithelial cells, as compared to age-matched WT controls (Figures 1A–1E, 1H). Similar to previous findings,^6^ sporadic insulin-expressing cells within CF pancreatic ducts were seen but were not present in WT pancreas (Figures 1F and 1G). Additionally, a subset of CF ductal cells expressed higher levels of acinar markers AMY2B^29^ and RNASE1^30^ (Figures S1A–S1D, S1G), but had reduced expression of the ductal specific marker HNF6^31^ (Figures S1E–S1G), as compared to WT controls. These findings support a change in CF ductal cell phenotype that appears similar to those observed in ADM and PDACs.

To delineate cell-autonomous alterations to the CF ductal cell phenotype, we used an approach previously developed for the expansion and polarization of WT and CF ferret pancreatic ductal cells (PDCs) in culture^16^ (Figure 1I). To obtain a uniform population of pan-cytokeratin-expressing ductal epithelial cells, primary cells were cultured for 10 passages prior to polarization at an air-liquid interface (ALI). We observed genotypic differences in mRNA expression for several ductal markers in proliferating PDCs and polarized pancreatic ductal epithelia (henceforth called PDE when speaking about ALI cultures), with *KRT7*, *CDH1*, and *SOX9* expression being elevated in CF and *HNF6* being lower in CF (Figure 1J). Additionally, PDX1 expression was uniformly elevated in CF in PDC and PDE cultures, as compared to WT controls (Figure 1J). Thus, *in vivo* changes in PDX1, SOX9, and HNF6 expression in CF pancreatic ducts were also observed at the mRNA level in PDC and PDE cultures.

### *CFTR*-KO PDE cultures adopt mesenchymal features following polarization

**Figure 2:**
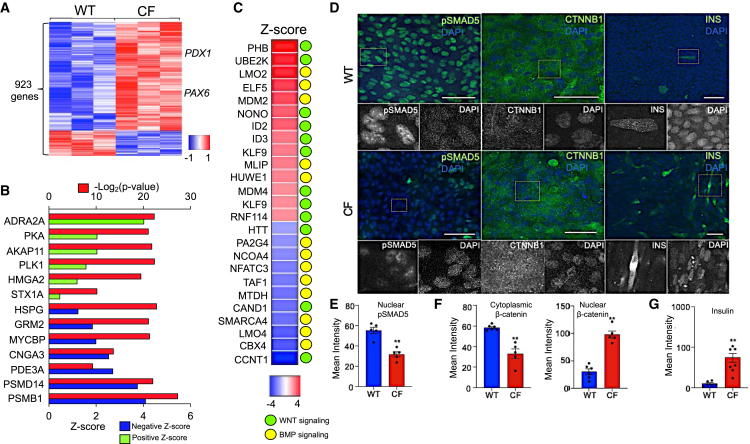
Transcriptional changes in CF PDE implicate WNT activation and BMP repression (A) The bulk transcriptomes of newborn WT and CF PDE cultures were sequenced. Heatmap shows differentially expressed genes following Benjamini-Hochberg correction (*p* < 0.05). (B) Upstream regulators of observed DEGs were obtained from IPA analysis. The *Z* scores and *p* values of the top regulators are shown. (C) Overlap between putative upstream regulators of DEGs found in PDE cultures and known regulators of pancreatic endocrine progenitors specific genes. Activation *Z* scores are displayed in the heatmap. Association of the candidate upstream regulators with WNT or BMP signaling is indicated on the right. (D) Whole-mount localization of pSMAD5 (BMP regulator) and nuclear CTNNB1 (β-catenin, WNT regulator) with insulin in WT and CF PDE cultures. Images were obtained on confocal microscope Zeiss 880 at 20X magnification and processed for maximum intensity projection. (E) Quantification of nuclear pSMAD5 in WT (*n* = 5 donors) and CF (*n* = 5 donors) PDE cultures. (F) Quantification of cytoplasmic and nuclear CTNNB1 in WT (*n* = 6 donors) and CF (*n* = 5 donors) PDE cultures. (G) Quantification of insulin expression in WT (*N* = 6 donors) and CF (*N* = 7 donors) PDE cultures. Bar plots in (E–G) show mean intensity of expression from 3 transwells per donor +/− SEM. Significance was calculated using nonparametric Mann-Whitney t test (∗∗*p* < 0.01).

To further clarify genotype-specific changes in ductal cell phenotype, we performed bulk RNA sequencing (RNA-seq) on WT and CF PDE cultures. Differential gene expression analysis followed by gene ontology (GO) and upstream regulator analysis inferred alterations to signaling pathways in CF PDE cultures (Figures 2A–2C and S2). Of the 16,574 expressed genes, 923 genes were differentially expressed after benjamini hoschberg (BH) correction (corrected *p* value <0.05) in CF PDE cultures (Figures S2A, 2A, and Table S1A). Of the differentially expressed genes (DEGs), 27 genes were upregulated (Log_2_FC > 2) in CF PDEs and 9 genes were downregulated (Log_2_FC < −2) (Table S1A). GO term analysis of DEGs revealed pathways involved in *Mesenchyme Differentiation and Development*, *Positive regulation of Cell Migration*, and *Cell-Cell Junction Organization*, and *Protein Localization in Membrane* (Figure S2B and Table S2A), suggesting CF PDEs retain features associated with a loss in epithelial characteristics. In support of this hypothesis, the upregulation of collagen genes plays key role during EMT^32^ and 10 collagen genes were upregulated (Log_2_FC > 2) in CF PDEs (Table S1A). However, genes known to repress or activate EMT, such as *TMEM45B*, *ECRG4*, *CHRDL1*, and *PCDH10*, were also among the most highly upregulated genes in CF PDE, suggesting a transitioning cellular state (Table S1A). *PDX1* expression was significantly (*p* = 4.44E−04) increased 6.6-fold in CF PDEs (Table S1A). Notably, BMP signaling inhibitor (*CHRDL1*) and WNT signaling activator (*TRIM14*) were upregulated 5.7-fold and 5.6-fold in CF PDEs (*p* < 0.05), respectively (Table S1A), indicating potential repression of BMP and activation of WNT signaling.^33^^,^^34^ This was supported by GO term and upstream regulator analysis of DEGs where *cell differentiation*, *mesenchyme differentiation*, *cell migration*, and EMT pathways including WNT and TGF-β were activated and BMP signaling was inhibited (Figures S2B and S2C, Figures 2B and 2C, Tables S1B and S1C, Tables S2A–S2F).

The putative upstream regulators of the DEGs in CF PDEs that were associated with mesenchymal transition were found to include WNT and BMP signaling regulators (Figure 2C and Table S1B). For example, activation of upstream regulator *ID2* (*p* = 4.7E−6; *Z* score = 3.0) has been correlated with increased WNT signaling cancer stem cells.^35^ Upstream WNT regulator MYCBP^36^^,^^37^ was also activated (*p* = 3.8E−7; *Z* score = 2.0), while inhibition of BMP receptor 2 (BMPR2) was observed (*p* = 2.54E−6; *Z* score = −4.0) (Figure 2B and Table S1B). Similarly, we observed activation of UBE2K (*p* = 1.01E−4; *Z* score = 3.973), a target of WNT signaling that aids the progression of EMT (Figure 2C, Tables S1B and S1C),^38^ whereas upstream regulated NFATC3 was inhibited (*p* = 2.22E−7; *Z* score = −3.0)—a transcription factor activated by BMP signaling that is known to prematurely disrupt mesenchymal transition (Figures 2C, Tables S1B and S1C).^39^^,^^40^ Similarly, BMP signaling activator SMARCA4, a factor known for maintenance of epithelial-like gene signatures,^41^ was inhibited (*p* = 3.05E−6; *Z* score = −3.5) (Figures 2C and Table S1C). Overall, the enhanced mesenchymal signatures in the bulk transcriptome of CF PDEs implicated changes to signaling pathways (BMP, TGF-β, and WNT) that also play important roles in cell fate decisions during pancreatic development and disease (Figures S2B, S2C, 2B, and 2C).

Despite *in vitro* culture for 10 passages prior to polarization at an ALI, genes associated with inflammation remained upregulated in CF PDEs, including the inflammasome-associated *PYCARD* (Log2FC = 5.62, *p* = 2.28E−08)^42^ and activator of nuclear factor κB (NF-κB)/tumor necrosis factor alpha (TNF-α) signaling in pancreatic cancer *VRK2* (Log2FC = 2.25, *p* = 8.6E−04)^43^ (Table S1A). Furthermore, many chromatin remodeling genes were lower in CF PDEs (Figure S2E and Table S1A) including *CBX2*,^44^ *KDM6A*,^45^ and *H1-0*,^46^ suggestive of an altered chromatin state (Table S2G).

### TGF-β1, BMP, and WNT signaling are altered in *CFTR*-KO PDCs *in vivo*

We assessed whether the inferred transcriptional changes in BMP and WNT signaling of CF PDE cultures reflected those observed *in vivo*. Active BMP signaling is associated with increases in nuclear phosphorylated suppressor of mothers against decapentaplegic (pSMAD), and thus we hypothesized that pSMAD levels would be lower in CF ductal epithelia. Indeed, nuclear pSMAD5 levels in CF PDEs (Figures 2D and 2E) and CF pancreas (Figures S3G, S3H, and S3L) were ∼2-fold lower than WT counterparts, supporting the hypothesis that BMP signaling is suppressed in CF ductal cells.

Accumulation of nuclear β-catenin is associated with activation of canonical WNT signaling, and nuclear β-catenin is elevated in CF human pancreatic ducts.^17^ Similarly, CF PDEs demonstrated an increase in nuclear β-catenin (∼2-fold) and decrease (∼3-fold) in cytoplasmic β-catenin (Figures 2D–2F). To substantiate that WNT signaling is activated in the CF ferret pancreas, we evaluated localization of *WNT7A*,^47^ which was previously shown to be upregulated in human CF PDCs by scRNA-seq.^17^ Indeed, clusters of *WNT7A*-expressing cells were observed in ∼2-month-old CF pancreatic ducts but were largely absent in WT controls (Figures S3C, S3D, and S3J). Similarly, AXIN2 expression, a marker of WNT activation,^47^ was increased (∼18-fold) in CF ductal pancreatic epithelium (Figures S3A, S3B, and S3I). These findings support the hypothesis that WNT signaling is activated in CF ductal cells *in vitro* and *in vivo*.

TGF-β1 plays major roles in EMT during development and disease and similarly promotes endocrine differentiation during pancreatic development.^48^^,^^49^ Consistent with CF PDE gene signatures, *TGFB1* mRNA expression was significantly (*p* < 0.05; ∼100-fold) higher in 2-months-old CF ferret ductal epithelium as compared to WT controls (Figures S3E, S3F, and S3K). Given that TGF-β1 and EMT are involved in endocrine progenitor fate initiation,^48^ we stained WT and CF PDEs for insulin following polarization. CF PDEs retained greater numbers of insulin-expressing cells compared to WT (*p* < 0.01) (Figures 2D and 2G). As previously shown,^6^ CF ferret pancreatic ductal epithelium also contained sporadic insulin-expressing cells *in vivo*, which was not observed in WT pancreas (Figures 1F and 1G). Collectively, the phenotypic changes observed in CF PDEs *in vitro* and pancreatic ducts *in vivo* appeared similar to those typically associated with multipotent ductal progenitors during pancreatic development.

### The epigenome of CF PDEs is reprogrammed with multipotent progenitor signatures

**Figure 3:**
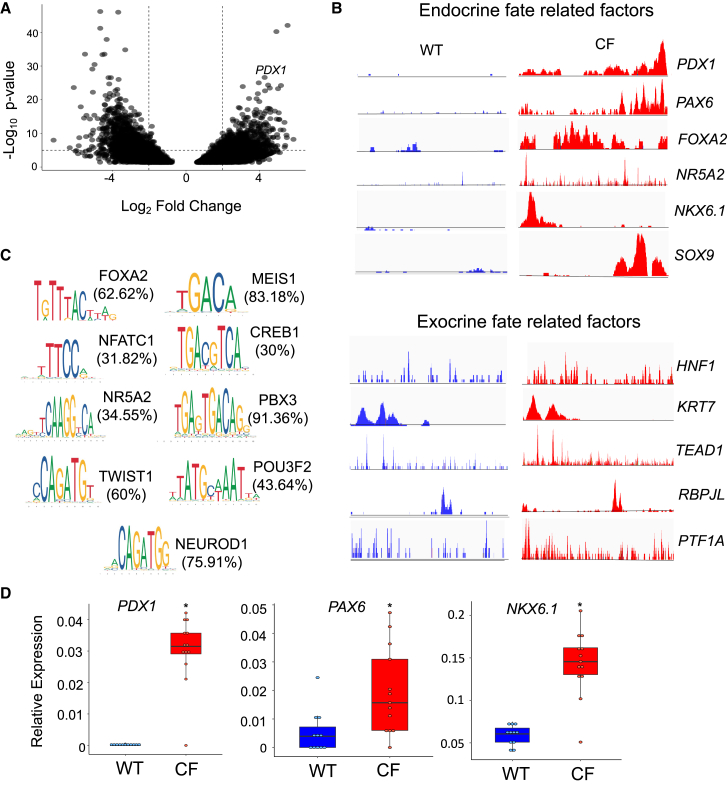
Altered epigenome in CF PDE cultures leads to greater accessibility of endocrine lineage-related genes (A) Differentially open regions (DORs) in the genome of CF relative to WT PDE cultures are shown in the volcano plot. Regions in the genome with −2< or >2 Log2FC read alignment were considered differentially open between genotypes. (B) Histograms of the number of reads aligned to genomic loci of endocrine fate-related factors and exocrine fate-related factors. (C) Analysis of motif enrichment (AME) for TF binding site motifs in the open regions of the CF PDE genome. Enriched motifs of endocrine fate-related TFs are shown. The percentage of open regions with the shown motifs is given in parentheses. (D) RT-qPCR quantification of endocrine fate-associated gene expression in WT (*n* = 11 cultures from 6 donors) and CF (*n* = 13 cultures from 6 donors) PDE cultures. Boxplots indicate mean relative expression +/− SEM. Significance was calculated using nonparametric Mann-Whitney t test (∗*p* < 0.05).

The reduced expression of chromatin regulatory genes in CF PDEs suggested epigenomic modifications could be responsible for altering the CF ductal cell phenotype. To this end, we used bulk assay for transposase-accessible chromatin using sequencing (ATAC-seq) to evaluate differentially accessible chromatin in WT and CF PDEs following 14-day of polarization. Approximately 13,000 regions were significantly (*p* < 0.05) differentially accessible as determined by DiffBind analysis (Figure 3A) (Table S3A). Genomic loci of endocrine progenitor transcription factors *PDX1* (Log_2_FC = 4.3, *p* = 2.94E−27) and *PAX6* (Log_2_FC = 2.25, *p* = 1.9E−05) among others were significantly more open in CF as compared to WT PDE cultures (Figure 3B and Table S3A), consistent with enhanced *PDX1* expression in CF PDE cultures (Figure 1J and Table S1A). Histone-modifying genes described to suppress endocrine progenitor specification,^50^^,^^51^^,^^52^ including histone methyltransferase *EZH2* (Log_2_FC = −2.00, *p* = 0.000901), histone deacetylases *HDAC4* (Log_2_FC = −3.29, *p* = 8.46E−16), and *HDAC9* (Log_2_FC = −2.18, *p* = 1.35E−5), were differentially closed in CF PDE cultures (Table S3A). Disruption of these genes in mice^51^^,^^52^ or treatment with HDAC inhibitors^53^ promotes endocrine specification, whereas overexpression of HDACs inhibits specification of beta and delta cells.^52^ With the exception of *SOX9*, which was more open in CF PDEs and has roles in maintaining both bipotent pancreatic progenitors and pancreatic ductal identity,^21^^,^^54^ the chromatin of exocrine fate-related factors were relatively unchanged in CF vs. WT PDE cultures (Figure 3B).

While SOX9 is a marker of ductal cell lineages in the adult pancreas, during pancreatic development SOX9 induces expression of *NEUROG3*—the transcription factor required for specification of endocrine cell lineages.^54^ SOX9 has also been described to initiate acinar to ductal reprogramming and maintain the pancreatic multipotent and bipotent progenitor pool.^21^ Thus, open chromatin at the *SOX9* locus (Figure 3B) and enhanced *SOX9* expression in CF PDCs and PDE (Figure 1J) are also consistent with expansion of ductal-derived progenitors in CF ductal epithelium. Additionally, *NR5A2* (Log_2_FC = 3.9, *p* = 3.2E−08), a trunk-specific transcription factor known for its role in maintaining the progenitor population^55^ and generation of acinar cells during development,^56^ and *PROM1* (Log_2_FC = 2.94, *p* = 0.00474), a duct epithelial progenitor marker,^57^ were both differentially open in CF PDE (Table S3A). These findings suggest the presence of a mixture of progenitor cell phenotypes in CF PDE capable of exocrine and endocrine lineage specification.

To gain a better understanding of the altered transcriptional landscape in CF PDE, the differentially open genomic regions were evaluated for enrichment of transcription factor binding motifs using analysis of motif enrichment (Figure 3C and Table S3B). This analysis revealed significant enrichment of binding sites within open chromatin for several transcription factors involved in regulating pancreatic endocrine and exocrine fate including *FOXA2* (enriched in ∼62% of the sequences),^58^ *NEUROD1* (enriched in ∼75% of the sequences),^59^ and *NR5A2* (enriched in ∼35% of sequences)^55^ among others (Table S3B). GO term analysis of enriched motifs indicated activation of *endocrine developmental program*, with increased expression of endocrine lineage-related factors found in CF PDEs (Figure S2D and Table S2H). As expected, pancreatic progenitor and endocrine specification transcription factors *PDX1* (100-fold, *p* < 0.001), *PAX6* (4-fold, *p* < 0.05), and *NKX6.1* (5-fold, *p* < 0.05) were expressed at significantly higher levels in CF PDE cultures (Figure 3D). These results further substantiate the observed transcriptional changes in CF PDCs that implicate altered potential for differentiating toward exocrine and endocrine lineages.

### WT and CF PDCs contain divergent and distinct lineages during polarization and differentiation

To better understand the genotype-specific differences in fate potential of PDCs, we performed scRNA-seq on neonatal WT and CF PDCs during polarization and differentiation at an ALI. The WT and CF PDEs were sequenced on 2, 5, 7, and 9 days of polarization (Figure S4A). Three WT and three CF donors with an average difference in *PDX1* expression of ∼1,600-fold in PDC proliferating culture, and ∼100-fold following polarization, were pooled and used for scRNA-seq (Figures S4B and S4C). An average of ∼6,000 cells per sample were sequenced. Only cells with mitochondrial RNA less than 20% of the total RNA were included in the subsequent analyses. Principal-component analysis followed by clustering using Seurat identified prominent pancreatic cell states at all stages of differentiation.^60^ Clustifyr was used to assign cell states to the predicted clusters^61^ by correlating average expression of each gene to previously published single-cell expression datasets.^62^^,^^63^^,^^64^^,^^65^^,^^66^ The cell state with the highest correlation was assigned to the cluster.

**Figure 4:**
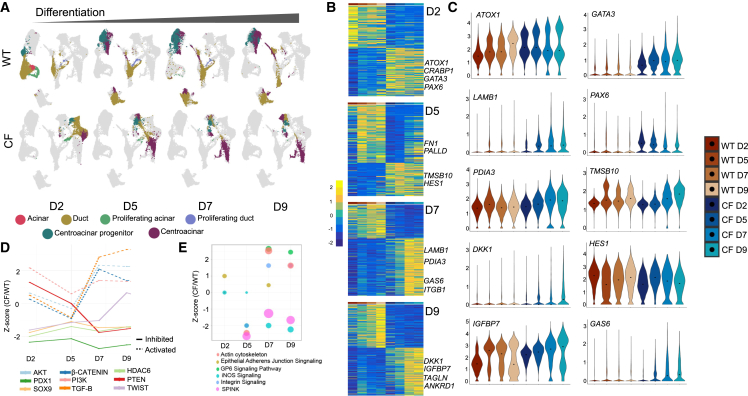
Single-cell transcriptomes of actively differentiating PDCs reveal acquisition of an epithelial-to-mesenchymal transitional phenotype in CF PDE cultures (A) WT and CF PDE on day 2 (D2), day 5 (D5), day 7 (D7), and day 9 (D9) of differentiation at ALI underwent 10× single-cell RNA sequencing (scRNA-seq). Shown are uniform manifold approximation and projections (UMAPs) of cell types at each time point of differentiation; cell markers for classification used a combined mouse and human scRNA-seq pancreatic datasets. (B) Heatmaps of DEGs in CF relative to WT PDE cultures at each time point. Genes relevant to epithelial-to-mesenchymal transition (EMT), diabetes, and cystic fibrosis are highlighted. Time point of differentiation is shown in the color-coded panel on top of each heatmap (using the legend shown in C). (C) Violin plots of DEGs found in WT vs. CF PDE cultures depicting Log2(TPM) expression level at each time point of differentiation. (D) Putative upstream regulators of the DEGs in CF PDE cultures. Activation *Z* score of candidate regulators is shown at each time point of differentiation. *Z* scores >2 indicate activation (dotted lines) and <−2 indicates inhibition. (E) Activation *Z* scores of IPA pathways altered in CF relative to WT PDE cultures at each time point of differentiation.

The predicted identities of the cell clusters were acinar, ductal, proliferating acinar, proliferating ductal, centroacinar progenitor, and centroacinar (Figures S4E, S4F, Figure 4A, and Tables S4A–S4F). Clusters with differentially upregulated expression of *ADIRF*,^67^ *CAPG*, *S100A10*^63^, and keratins (*KRT5*, *KRT15*, *KRT19*) were assigned ductal identity (Figures S4E, S4F, and Table S4B).^65^ Enrichment of IPA pathway analysis terms for this ductal cluster included *branching morphogenesis of epithelial tube*, *negative regulation of cell migration*, and *columnar/cuboidal epithelial cell differentiation*. Together with ductal expressed markers *ADIRF*,^67^ *ADIRF*, and *S100A10*,^63^ differential expression of cell-cycle markers like *CCNA2*, *CCNB1/2*, *CDC20*, and *CDCA2* was used to classify proliferating ductal cells (Figures S4E, S4F, and Table S4D). Acinar cells were marked by *LDHA*, *ASNS*, *PRDX2*, *PRDX4*, *FKBP4*, *FKBP11*, and *NUPR1*, which were also found as acinar markers in previous single-cell studies^63^ (Figure S4F and Table S4A). The lack of expression of prominent acinar markers like *AMY2B* is believed to be due to the ductal origin of these acinar-like cells (Figures S4E, S4F, and Table S4A). Clusters differentially expressing the acinar genes *PRDX2*, *PRDX4*, and *FKBP11*,^63^ as well as cell-cycle regulatory genes like *CCNB1/2*, *CDC20*, and *CDCA2*, were assigned as proliferating acinar cells (Figures S4D–S4F and Table S4C). Clusters called centroacinar cells had a combination of ductal (*KRT7*, *KRT16)* and the centroacinar-enriched genes (*WSB1*, *PROM1*, *S100A6*, *S100A4*) previously reported by scRNA-seq^65^ (Figures S4F and Table S4F). Finally, clusters assigned centroacinar progenitor cell identity was characterized by *ALDH1A1* and *BCL2A1* enriched expression^68^^,^^69^ together with expression of multipotency regulator proteins like *KLF4* and *TFF3*,^65^^,^^70^ progenitor-like pancreatic duct epithelial marker *TSPAN8*,^65^ mucins (*MUC1*, *MUC13*, *MUC16*, *MUC4*, *MUC5AC*, *MUC5B*, *MUC20*), and exocrine markers *CEACAM* (Figures S4E, S4F, and Table S4E).

**Figure 5:**
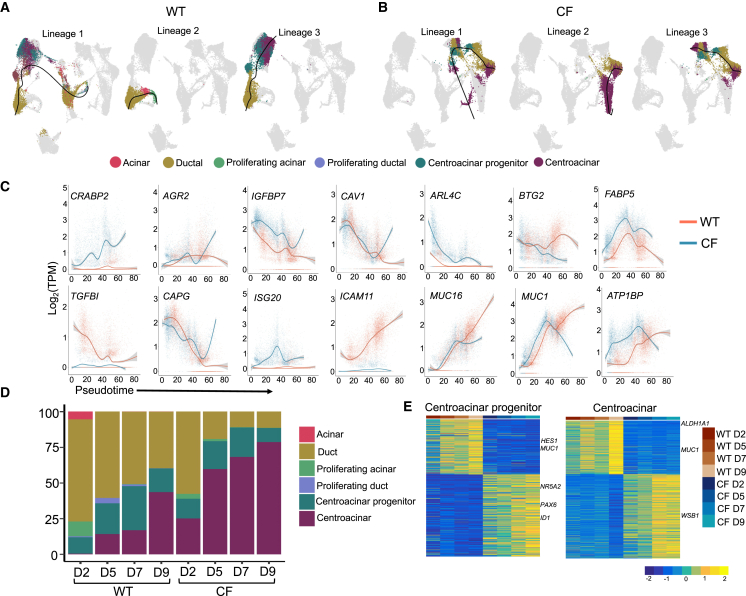
CF PDE cultures predominantly differentiate to centroacinar cells (A and B) Lineage trajectories observed in differentiating (A) WT and (B) CF PDE cultures using slingshot pseudotime ordering. (C) Variable expression profile of genes associated with centroacinar progenitors and centroacinar cell development in CF and WT PDE lineages. (D) Relative proportions of cell types in WT and CF PDE cultures at different time points of differentiation. A higher relative proportion of centroacinar cells is observed in CF PDE cultures. (E) DEGs in centroacinar progenitor and centroacinar cells generated from WT and CF PDE cultures at day 9 of differentiation.

The WT and CF PDEs had variable differentiation trajectories following polarization with significant genotype-specific differences in the derived cell states. WT PDEs were composed of predominantly ductal cells on day 2 and differentiated to a mixture of ductal and centroacinar cells by day 9 (Figures 4A and 5D). CF PDEs were also predominantly composed of duct cells on day 2 and by day 9 were predominantly composed of centroacinar cells (Figures 4A and 5D). Differential gene expression analysis between the two genotypes at each time point showed presence of signatures that altered during the course of differentiation. For example, *PAX6* was differentially enriched in CF PDEs on day 2 and higher in CF PDEs relative to WT PDEs at all time points (Figures 4B, 4C and Tables S5A–S5D), suggestive of endocrine differentiation^71^ and/or a phenotype similar to early duct/endocrine progenitors.^72^^,^^73^ Increased *HES1* in day-5 CF PDE was associated with the expansion of centroacinar cells^74^ and centroacinar progenitor cells on day 7 for which *HES1* has known roles^75^^,^^76^ (Tables S6A–S6F). *SPP1* is a marker for undifferentiated pancreatic progenitors^65^ and was elevated in CF PDE at day 7 to day 9 (Figures 4B and 4C, Tables S5C–S5D, and Tables S6A–S6F).

### CF PDEs acquire cell migration-associated markers during differentiation

Putative upstream regulators of the scRNA-seq DEGs in CF PDEs indicated the activation of AKT and PI3K and the repression of PTEN at day 5–9 (Figure 4D; Tables S7A–S7D). Epithelial identity is closely associated with cellular polarity with defined apical and basolateral domains. Cytoskeletal remodeling alters the distribution of epithelial polarity complex proteins and can change cellular identity.^76^ A known cell migration-inducing factor integrin subunit beta (ITGB) was upregulated in CF PDEs (Tables S5C, S5D, S6C, and S6D).^77^^,^^78^ Migration is associated with loss of adherence junctions, which eliminates apical-basal polarity and initiates front-rear polarization.^79^ Consistent with this, GAS6, a protein that associates with AXL and induces migration through ERK signaling, was upregulated in CF PDE (Tables S5C, S5D, Tables S6C, and S6D).^80^ Similarly, LAMB1 important for basement membrane formation and for induction of cell migration by the ERK pathway,^81^ was higher in CF PDEs (Tables S5C, S5D, S6C, and S6D). Substantiating this, PTEN (*p* = 2.30E−10) and twist related protein (TWIST) (*p* = 1.65E−13) were shown to be upstream regulators of the observed DEGs at each time point (Tables S7A–S7D and Figure 4D).

PTEN dephosphorylates PIP3 only when transiently bound to cell membrane. This restricts PTEN activity spatially and ensures increased density of PIP3 on the apical membrane, which is important for apical-basal polarity.^25^^,^^82^ Likewise, TWIST is necessary for cytoskeletal rearrangement when cells lose apical-basal polarity and acquire invasiveness during EMT.^83^ Activated β-catenin signaling was identified as an upstream regulator of CF ductal cell phenotype (Figure 4D and Tables S7A–S7D) and was supported by enhanced nuclear accumulation of β-catenin *in vivo* (Figures 2D and 2F). Furthermore, β-catenin depletion from adherence junctions and cytoplasm is a prominent mechanism for loss of epithelial polarity.^84^ Pathway analysis of CF PDE DEGs also included *integrin signaling* and *actin cytoskeleton* initiating on day 7 (Figure 4E and Tables S7E–S7H). GP6 signaling is also necessary for cellular migration^85^ and was also an upstream regulator activated in CF relative to WT on day 7 and day 9 (Figure 4E and Tables S7E–S7H). Taken together, upregulation of pathways that contribute to loss in epithelial polarity and cell fate transitions was observed in CF PDE cultures.

Multiple ductal subpopulations were identified in both WT and CF PDE (Figure S6A and Tables S8A–S8F) each of which had unique changes in gene expression (Figure S6B and Tables S8A–S8F); GO term analysis of the differential gene expression patterns from WT and CF PDE ductal cells (Figures S6C and S6D) indicated inhibition of BMP signaling and activation of PI3K-AKT signaling, which was similar to the analysis on bulk RNA-seq of WT and CF PDE.

### WT and CF PDE cultures contain progenitors with unique trajectories during polarization and differentiation

In order to determine the temporal dynamics of genotypic changes in PDE phenotype following polarization, the sequenced transcriptomes were ordered on pseudotime using Slingshot. Slingshot first determines the number of lineages and branching points for each trajectory and then estimates cell-level pseudotime variable for each lineage.^86^ Using this method, three principal lineages were predicted for both WT and CF PDE cultures, with the ductal cells as the starting point for WT and centroacinar cells for the starting point of CF for all predicted lineages (Figures 5A and 5B). In the WT PDEs, the ductal cells transitioned through a centroacinar-like state and differentiated mostly to acinar or ductal cells, whereas the CF centroacinar cells differentiated primarily to centroacinar progenitors and ductal-like cells (Figures 5A and 5B). Given the genotype-dependent discrete clustering observed for the derived cell states, we sought to understand the genetic differences between WT and CF-derived centroacinar, centroacinar progenitors, acinar, and ductal cells. Particularly, we characterized the difference in WT and CF-derived centroacinar and centroacinar progenitor cells in WT and CF lineage 3 (Figures 5A and 5B).

Genes differentially expressed in the WT and CF pseudotime lineage 3 trajectory are shown in Figure 5C. Retinoic acid signaling has been shown to be important for centroacinar cell differentiation and maintenance,^87^^,^^88^ and cellular retinoic acid binding protein 2 *(CRABP2)* gene expression was upregulated over time in the CF trajectory during differentiation (Figure 5C). Similarly, *AGR2*, which is known to induce translocation of epidermal growth factor receptor (EGFR) to the membrane during pancreatic regeneration and for proliferation of progenitors *in vitro*,^89^^,^^90^ also increased with time of CF PDE differentiation (Figure 5C). Genes associated with fibrosis (*FABP5*) and activation of stellate cells (ISG20)^88^^,^^91^^,^^92^ were also higher in the CF lineage (Figure 5C). ARL4C, which is activated by WNT signaling,^93^^,^^94^ was higher in CF at all stages of differentiation (Figure 5C). Notably, *MUC16* and *ICAM1* were lower in CF and are genes shown to be downregulated during EMT (*MUC16*).^95^ Likewise, cancer biomarker (*ICAM1)* was downregulated in CF PDEs^96^^,^^97^ (Figure 5C). Quantification of relative proportions of predicted cell types at each time point of differentiation showed an increase in centroacinar cells in CF PDEs from ∼25% (day 2) to ∼80% (day 9) (Figure 5D). By contrast, centroacinar cells were absent from WT PDE cultures on day 2 but rose to ∼40% of the culture by day 9.

### PTEN-associated signaling pathways are upregulated in CF PDE centroacinar cells

During late phases of pancreas development, Notch and its target gene Hes1 promote ductal differentiation from exocrine-restricted progenitors and then maintenance of centroacinar cell fate in the mature pancreas.^74^^,^^98^ Notch/HES1 also repress cell fate commitment by multipotent and bipotent pancreatic progenitors during development,^99^ and thus lower *HES1* expression in CF-derived centroacinar progenitors is consistent with a more pliable progenitor cell state (Table S6E and Figure 5E). Furthermore, higher expression of *PAX6*, *ID1*, and *ID3* in CF centroacinar progenitors supports known functions in ID protein maintenance of a stem cell state and PAX6 involvement in endocrine cell specification^73^^,^^100^^,^^101^ (Tables S6E and Figure 5E). Additionally, CF centroacinar progenitors appeared more proliferative given the upregulation of cell-cycle genes CCND1 and CCND2 (Tables S6E and Figure 5E).

Like CF centroacinar progenitors, DEGs in CF centroacinar cells included upregulation of endocrine lineage genes ID2 and ID3 (Table S6E and S6F), but also enhanced expression of WNT mediator CTNNB1^102^ (Table S6F) and PI3K signaling subunit PIK3R1, and downregulation of ductal genes like MUC1 and KRT8 (Tables S6E and S6F and Figure 5E). Upstream regulator analysis on DEGs between WT and CF-derived centroacinar cells and centroacinar progenitors showed upregulation of TGF-β (*p* = 2.67E−55), WNT (*p* = 1.37E−37), and AKT (*p* = 4.41E−18) signaling (activation *Z* score >2) and inhibition of PTEN (*p* = 6.34E−21) only in CF centroacinar cells (activation *Z* score < −2) (Tables S9A and B) (Figures S5A and S5B). AKT signaling-associated genes *TSC2* and *EIF4E*, both regulators of cell cycle and mTOR-related tissue regenerative mechanisms,^103^^,^^104^ were higher in CF centroacinar cells (Table S9C). Enhanced expression of the PTEN inhibitor *CREB3L2* was observed in CF centroacinar cells (Table S9D). PTEN signaling contributes to epithelial polarity,^25^ and loss of PTEN is thought to promote PDAC from centroacinar cells and EMT.^105^ Furthermore, *PDPK1* (PDK1), a gene required for expansion of exocrine and endocrine pancreatic progenitors during development,^106^ was also elevated in CF centroacinar cells (Table S9D). TGF-β signaling is known for its roles in EMT during the duct to endocrine transition of pancreatic development,^49^ and *THBS1*, a TGF-β signaling target gene and EMT activator,^107^ was upregulated in CF centroacinar cells (Table S9F). Upstream activators associated with WNT signaling in CF centroacinar cells (Figures S5A and S5B) included enhanced expression of *LEF1* and *TCF7L2* relative to WT centroacinar cells (Table S9E), which are known to be associated with enhanced proliferation in pancreatic cancer.^108^ Taken together, the altered phenotype in CF PDEs centered around TGF-β-mediated inhibition of PTEN signaling, activation of PTEN downstream target AKT, and WNT activation likely through AKT-mediated inhibition of GSK3B. These scRNA-seq studies thus confirmed many of the findings in bulk RNA-seq and ATAC-seq and implicate these pathways as mediators of cellular programs governed by PDX1 and global changes in the epigenetic landscape of CF PDEs.

### Cell-autonomous reprogramming of pancreatic ductal epithelium occurs in the absence of CFTR protein not function

**Figure 6:**
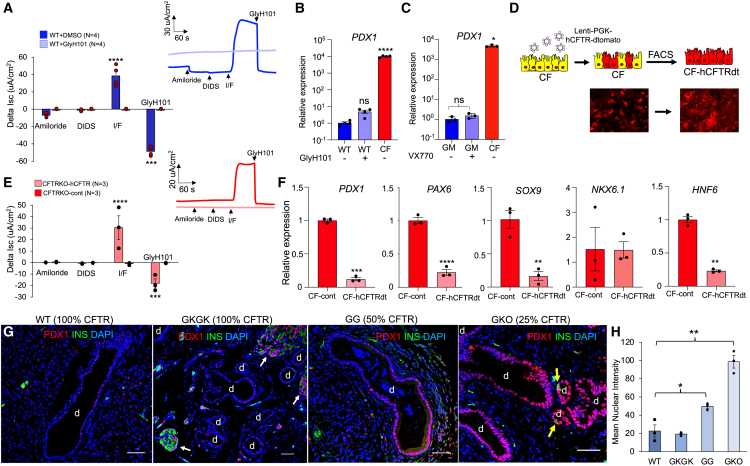
CFTR protein presence, but not function, regulates PDX1 expression (A) Short circuit current measurement in WT PDE cultures differentiated in the presence of CFTR inhibitor GlyH101 or vehicle (DMSO). Responses to sequential addition of amiloride, DIDS, IBMX/Forskolin (IF), and GlyH101 are shown (*n* = 4 donors with 2 cultures averaged per donor). Inset is a representative current trace for each condition. (B) RT-qPCR quantification of *PDX1* expression under the conditions show in (A). *PDX1* expression in CFTR-KO PDE cultures without GlyH101 is shown for comparison (*n* = 4 donors with 3–4 cultures combined for RNA). (C) *PDX1* expression in PDE cultures derived from ferret PDCs with homozygous CFTR-G551D germ line and differentiated in the presence of VX-770 (+) or DMSO (−) (*n* = 3 donors with 3–4 cultures combined for RNA). *PDX1* expression in CFTR-KO PDEs is shown for comparison (*n* = 3 donors with 3–4 transwells combined for RNA). *PDX1* expression is normalized to that of (B) WT (−) or (C) G551D (−). (D) Schematic of human CFTR (hCFTR) complementation in CFTR-KO PDCs using a lentiviral vector that also expresses tdTomato. A vector expressing just tdTomato was used as a control (cont). FACS-enriched tdTomato-positive cells were used to generate PDE cultures. Representative images of PDC cultures before and after FACS enrichment are shown below the schematic. (E) Short circuit current measurements of PDE cultures-derived lentiviral transduced CFTR-KO PDCs expressing hCFTR/tdTomato or tdTomato alone. Responses to sequential addition of amiloride, DIDS, IBMX/Forskolin (IF), and GlyH101 are shown (*n* = 3 donors). Inset is a representative trace of current for each condition. (F) RT-qPCR quantification of ductal (*SOX9*, *HNF6*) and endocrine (*PDX1*, *PAX6*, *NKX6.1*) genes expression from the conditions in (E) (*n* = 3 donors with 2 cultures combined for RNA). Expression levels are normalized to CFTR-KO tdTomato PDE cultures. (G) Immunofluorescence images of PDX1 and INS expression in 2-month-old WT ferrets and *CFTR* mutants ferrets with variable expression of CFTR protein (percent CFTR expression is shown on top of each image). Images were obtained on confocal microscope Zeiss 880 at 20X magnification and processed for maximum intensity projection. Scale bars, 50 μm. (H) Quantification of PDX1 expression in ductal epithelium (*n* = 3 donors for each genotype). All graphs show the mean ± SEM. Significance was calculated using nonparametric Mann-Whitney t test (∗*p* < 0.05, ∗∗*p* < 0.01, ∗∗∗*p* < 0.001, ∗∗∗∗*p* < 0.0001).

Pancreatic pathology in CF is hypothesized to be caused by the lack of CFTR-mediated bicarbonate secretion, which lowers pH and leads to premature activation of pancreatic enzymes and the inflammatory destruction of acinar cells.^2^ Based on the transcriptional signatures of CF ductal epithelium, we hypothesized that ductal cell reprogramming was secondary to inflammatory-dependent expansion of a unique progenitor and/or CFTR-dependent channel functions (i.e., luminal pH regulation) that impact epithelial phenotype. To approach the later of these hypotheses, we asked whether inhibition of CFTR function in WT PDEs would impact *PDX1* expression (Figures 6A and 6B). Notably, continuous treatment of differentiating WT PDEs for 14-day with CFTR inhibitor GlyH101 inhibition did not significantly alter *PDX1* expression (Figure 6B), despite effective inhibition of CFTR-mediated chloride currents (Figure 6A). We have previously shown that CFTR-G551D ferret PDEs are responsive to the CFTR modulator VX-770, which restores channel gating.^109^ Thus, we next sought to evaluate whether VX-770 rescue of CFTR-G551D function in CF PDEs would inhibit *PDX1* expression. Contrary to our hypothesis, *PDX1* expression in CFTR-G551D PDEs was 1000-fold lower than CFTR-KO PDEs regardless of whether they were differentiated in the presence of VX-770 (Figure 6C). Given that CFTR-G551D PDC cultures were derived from ferrets with similar pancreatic pathology to CFTR-KO PDC cultures (i.e., untreated with VX-770), these findings suggested that the altered PDX1 phenotype of CFTR-KO PDCs and PDEs was not a direct consequence of the inflamed state from which the cells were derived or CFTR channel function.

Since CFTR-KO PDEs lack both CFTR function and protein presence on the membrane, we asked whether reconstituting the CFTR protein in CFTR-KO PDEs would restore *PDX1* expression to WT levels. CFTR-KO PDCs were genetically modified using lentiviral vectors to express h*CFTR*/tdTomato or tdTomato alone and then enriched for tdTomato-expressing cells by fluorescence-activated cell sorting (FACS) prior to plating at an ALI (Figure 6D). As expected, PDEs cultures generated from h*CFTR*/tdTomato-expressing PDCs generated CFTR currents that were significantly greater (*p* < 0.0001) than tdTomato-expressing controls (Figure 6E). Notably, complementation of CFTR expression led to reduced *PDX1*, *PAX6*, and *SOX9* expression toward that of WT but had no effect on NKX6.1 expression (Figure 6F). Collectively, these findings implicated the cell-intrinsic presence of the CFTR protein, not its function, as the root cause of altered CFTR-KO ductal cell phenotype.

We next sought to obtain *in vivo* data to support a correlation between CFTR presence in PDCs and PDX1 expression. To this end, we evaluated PDX1 expression in pancreata derived from a series of CF ferret models with CFTR genotypic variants that alter the abundance of CFTR protein. These models included ferrets harboring (1) a biallelic CFTR-G551D mutation that expresses 100% of WT CFTR (GKGK),^110^ (2) a hypomorphic biallelic G551D mutation with only 50% CFTR expression (GG) due to a neomycin selection cassette in neighboring CFTR intron,^109^ and (3) a compound heterozygote harboring one CFTR-KO allele^111^ and one hypomorphic G551D mutation^109^ with only 25% CFTR expression (GKO). Finding from these pancreata demonstrated that PDX1 expression in the ductal epithelium of GKGK animals was similar to WT, while in GG and GKO pancreata PDX1 expression increased in concert with the extent of hypomorphic *CFTR* expression (Figures 6G and 6H). These findings support the *in vitro* observations implicating a cell-autonomous process by which CFTR protein presence, not function, alters the observed PDX1 phenotype in CFTR-KO ductal cells.

### PTEN inhibition and WNT activation alter ductal cell phenotype

Our results indicate that PTEN signaling in CF PDEs is perturbed and is a major upstream regulator of their altered transcriptional signature. Apical CFTR has been previously shown to be associated with multiple membrane proteins, including PTEN, where CFTR serves as a membrane anchor and regulator of PTEN activity, and this regulation does not require a functional CFTR channel.^112^^,^^113^ This is postulated to be necessary to restrict PTEN activity to the apical membrane and establish the apical-basal axis for epithelial polarization.^25^^,^^114^ PTEN inhibits PIP2 to PIP3 conversion by PI3K, necessary for AKT phosphorylation and activation. Activated AKT inhibits GSK3B to stimulate WNT signaling by repressing GSK3B-mediated phosphorylation and degradation of beta-catenin. Hence, active PTEN inhibits downstream WNT signaling by de-repression of GSK3B activity.^115^ However, the loss of CFTR-PTEN complex impairs PTEN activity^112^ and thus could activate WNT signaling.

**Figure 7:**
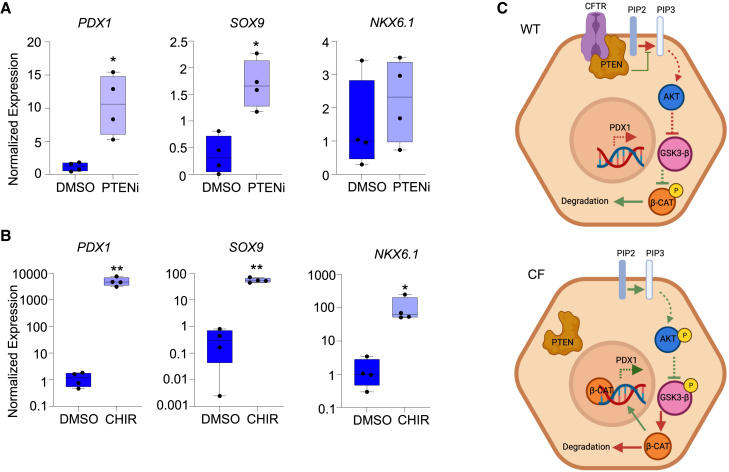
PTEN inhibition and WNT activation induce PDX1 expression in WT PDE (A and B) RT-qPCR quantification of *PDX1*, *SOX9*, and *NKX6.1* mRNA in WT PDEs exposed to PTEN inhibitor (A) (PTENi) and WNT agonist (B) (CHIR). Boxplots show the mean relative expression +/− SEM for *n* = 4 donors per condition with ≥3 PDE cultures analyzed per donor and averaged. Significance was calculated using nonparametric Mann-Whitney t test (∗*p* < 0.05, ∗∗*p* < 0.01). (C) Schematic of proposed model for CFTR/PTEN-mediated *PDX1* regulation. The reactions that are proposed to be active in the presence or absence of CFTR are shown by green arrows (activating) and lines with caps (inhibitory), whereas reactions that are suppressed are indicated in red arrows and lines with caps. Dotted arrows indicate presence of intermediate reactions that are not shown in the schematic.

Since the lack of CFTR protein leads to enhanced *PDX1* expression in CFTR-KO PDCs and PDEs, we hypothesized that a similar mechanism of CFTR-mediated PTEN inhibition was responsible for alterations in CFTR-KO ductal phenotype. To formally test this, we perturbed the PTEN pathway at two important nodes—PTEN and GSK3B. We polarized WT PDEs in the presence of PTENi or GSK3Bi and evaluated their effect of *PDX1* and related progenitor genes *SOX9* and *NKX6.1* (Figure 7). Repression of PTEN activity resulted in significant increases in *PDX1* (*p* < 0.05) and *SOX9* (*p* < 0.05), but not NKX6.1 (Figure 7A). While pancreas-specific knockdown of PTEN has been previously reported to elevate *PDX1* expression in ducts,^26^ its effect on *SOX9* expression has not been shown. However, increased SOX9 expression is consistent with EMT initiation and also associated with PTEN inhibition.^116^^,^^117^

Activation of WNT by inhibiting GSK3B led to significantly higher levels of *PDX1* (*p* < 0.001), *SOX9* (*p* < 0.05), and *NKX6.1* (*p* < 0.05) in WT PDEs, again mirroring the CFTR-KO PDE phenotype (Figure 7B). Non-canonical WNT signaling has been shown to induce *PDX1* expression and prime the foregut cells for pancreatic lineage and prevent liver differentiation.^118^ WNT signaling also increases SOX9 expression during ductal branching morphogenesis. Additionally, *PDX1* and *SOX9* have been described to act cooperatively for pancreatic lineage specification and pancreatic progenitor maintenance.^50^^,^^54^^,^^119^ Collectively, these data support a structural role for CFTR in the maintenance of ductal cell phenotype though the control of PTEN/GSK3B axis.

## Discussion

Here we demonstrate that PDX1-positive ductal progenitors expand within the CF pancreas in the setting of acinar cell loss. *In vitro* expansion and differentiation of these progenitors suggest they are centroacinar cell derived and harbor bipotent progenitor cell transcriptional signatures that are typically associated with the specification of exocrine and endocrine fates during pancreatic development. Notably, these altered properties of CF PDCs were dependent on the loss of the CFTR protein, but not function, and are phenotypically distinct from WT ductal cells. Inhibition of PTEN or GSK3β led to the activation of PDX1 expression in WT ductal epithelium, giving rise to a similar phenotypic state in the *CFTR*-KO pancreas with enhanced Wnt/β-catenin activation and PDX1 expression. These findings suggest that CFTR residence on the apical membrane of ductal progenitors regulates their cell fate and may have implications for disease phenotypes in people with CF that harbor CFTR mutations that produce no or little protein.

Changes in CF pancreatic ductal phenotype have not been carefully studied, largely because CF mouse models lack the pancreatic phenotype observed in people with CF. In contrast to mice, the CF ferret pancreas undergoes significant remodeling within the first two months of life involving islet destruction, inflammation, fibrosis, adipogenesis, and ductal hyperplasia.^6^^,^^11^^,^^12^^,^^109^^,^^120^ The observed increase in PDX1 expression of *CFTR*-KO pancreatic ducts was postulated to be either due to the altered signals from the remodeled pancreatic environment or due to a cell-autonomous change associated with ADM, as often observed in PDX1-positive PDAC caused by chronic pancreatitis.^23^ Our *in vitro* studies with *CFTR*-KO proliferating PDCs and differentiated PDE, which remove extrinsic signals from the CF pancreas, support cell-autonomous changes in ductal cell phenotypes that enhance PDX1 expression. However, we appreciate that the loss of cellular microenvironment in *in vitro* systems can diminish the impact of environmental factors found *in vivo* and understand that *in vitro* differentiation can mask physiological lineage dynamics. Hence, we have attempted to verify our *in vitro* findings *in vivo* but emphasize that our results warrant further cell-specific validation.

*PDX1* has a well-characterized developmental role in initiating pancreatic and beta cell differentiation.^18^ Given that *PDX1* marks multipotent progenitors in the developing pancreas, higher *PDX1* and *SOX9* expression in *CFTR*-KO PDEs suggested a similar cellular state might exist in CF pancreatic ducts.^28^^,^^119^^,^^121^ Furthermore, ∼2% of the differentially open regions in the *CFTR*-KO ductal genome have been previously shown to bind PDX1 in pancreatic progenitors,^122^ highlighting PDX1 prominence in establishing the CF phenotype. While PDX1 binds to a Swi/Snf chromatin-modifying complex to initiate pancreatic progenitor differentiation,^123^ the cooperative role of both PDX1 and SOX9 in establishing and maintaining the progenitor pool is well established.^50^ In the adult pancreas, centroacinar cells are progenitors marked by *SOX9* and *HES1* but express little to no PDX1.^21^^,^^75^^,^^105^^,^^119^ However, PDX1 expression is observed in centroacinar cells and ductal cells of PDAC,^23^ and the ductal pattern in early stage cancers is strikingly similar to that in *CFTR*-KO ducts. Lineage tracing aberrant PDX1 expression in the CF pancreas could provide a specific cellular context to changes in signaling.

Inactivation of pancreatic developmental marker loci like *PBX1* (Table S3A) in CF PDE, previously known to induce islet malformations and aberrant ductal morphogenesis during development,^124^ suggested potential loss in normal differentiation potential. However, increased accessibility of islet differentiation factors like *ISL1* and *PAX6* (Table S2A) infers a disposition to endocrine lineages. Taken together, CF PDE displayed a mix of epigenetic signatures of a primordial state with abnormal regenerative capability. Given that ATAC-seq was a bulk assay, multiple differentially accessible genes were not differentially expressed. However, in addition to *PDX1*, one of the genes that was differentially open and differentially expressed in CF PDEs was *CACNA2D1* (Tables S1A and S3A). Notably this gene encodes a calcium channel complex that enables influx of Ca^2+^ and when defective leads to impaired islet insulin secretion and diabetes.^125^

PTEN is known to play an important role in maintenance of epithelial polarity, EMT, and cancer progression,^24^^,^^82^^,^^117^ and the transcription signatures of these pathways were altered in *CFTR*-KO PDEs (Figure S2). The localization of PTEN at the apical membrane of epithelial cells maintains PIP2 density important for polarity maintenance, and the loss of PTEN promotes EMT and malignant transformation.^26^^,^^117^ Pancreas-specific PI3K activation via PTEN KO increases expression of pancreatic progenitor genes *PDX1* and *HES1* in pancreatic ducts of mice via centroacinar cell metaplasia.^26^ Our bulk RNA-seq (Figure S2) and scRNA-seq (Figure S5) on *CFTR*-KO PDEs demonstrated transcriptional changes consistent with EMT, suppression of the PTEN pathway, and centroacinar cell expansion. Inhibition of PTEN would be expected to increase nuclear β-catenin and Wnt signaling, and this is consistent with enhancement of WNT7A, AXIN2, and nuclear β-catenin in CF pancreatic ducts and/or PDEs (Figures S3, S2, and 2C). The physical association of PTEN with CFTR at the plasma membrane maintains PTEN activity in airway epithelia, and CFTR absence inhibits PTEN activity leading to the activation of NF-κB though PI3K/AKT activation of inhibitor of nuclear factor k (IKK).^112^^,^^113^ These findings are strikingly similar to ours studying *CFTR*-KO ductal epithelium, in that CFTR mutants with stable membrane expression failed to inhibit the PTEN pathway and subsequent signaling alterations. Furthermore, the level of CFTR protein expression from hypomorphic mutants correlated with the ductal cell activation of PDX1 expression *in vivo*.

This study comes with limitations. Further analysis is needed to validate the CFTR/PTEN interaction in PDE cultures and determine the cell types *in vivo* for which this occurs. Utilization of transgenic ferrets with varying abundance of CFTR protein on the membrane (including newly reported *CFTR*^F508del^)^126^ would enable these studies and provide a better understanding of the PDX1-CFTR/PTEN axis *in vivo* using molecular markers of functional changes obtained in this study. These additional *in vivo* studies would provide greater physiological context to this research, given that our *in vitro* studies exclude a significant number of cell-cell interactions (duct-adipocyte, duct-fibroblast, etc) that could impact cell fate *in vivo*. Lastly, it is now possible to fate map progenitors *in vivo* using multi-transgenic ferret models^127^; thus, with the appropriate cell-specific Cre driver it will be possible in the future to fate map these phenotypic changes and better understand their *in vivo* implications to CF disease progression.

Our findings have potential implications for people with CF harboring CFTR mutation that produces no or little mutant protein like F508del. One previous clinical study in a relatively small cohort of people with CF (average age of 26–27 years), found that 23% of F508del/F508del patients developed impaired glucose tolerance or CFRD, as compared to 8% of patients with one G551D allele and a second severe mutation.^128^ In comparison to our studies in ferrets, these findings would suggest that reduced CFTR protein at the membrane in ductal cells has a negative impact on progression of CFRD. Whether this altered human phenotype relates to altered phenotype of ductal cells in the CF pancreas remains to be determined.

We propose that loss of CFTR at the membrane of centroacinar cells represses PTEN leading to the activation of AKT, inhibition of GSK3β, nuclear accumulation of β-catenin, and activation of WNT signaling (Figure 7C). Supporting this model are CFTR complementation experiments in CF PDEs and PTEN and GSK3β chemical inhibition experiments in WT PDEs, which either reserve or promote CF-associated changes in *PDX1*, *SOX9*, *PAX6*, and/or NKX6.1 expression. Coupled with scRNA-seq experiments in actively differentiating PDE cultures, we conclude that CFTR plays a structural role in maintaining pancreatic ductal epithelial phenotype, the loss of which leads to expansion of pancreatic progenitor cell state that is closely related to centroacinar cells.

### Limitations of the study

In this study we show that CFTR presence on the membrane regulates PDX1 expression in PDCs via GSK3β activity. This involves contact-based inactivation of PTEN due to lack of/reduced CFTR presence on the cell membrane. We believe mechanistic validation of CFTR-PTEN interaction regulating PDX1 in genotypes with varying abundance of CFTR protein on the membrane would provide better understanding of the PDX1-CFTR/PTEN axis. Additionally, cell type specificity of the PDX1-CFTR/PTEN axis *in vivo* would have provided more physiological context to the study. Furthermore, in regards to the *in vitro* ductal cell polarization system, we appreciate that it removes cellular environment-related changes that might contribute to PDX1 regulation in CF duct epithelium. We understand that the *in vitro* system described in this study can also mask differentiation phenotypic data found *in vivo*.

## Resource availability

### Lead contact

Further information and requests for resources and reagents should be directed to and will be fulfilled by the lead contact, John F. Engelhardt (john-engelhardt@uiowa.edu).

### Materials availability

This study did not generate new unique materials.

### Data and code availability

• All data generated for this paper will be shared by the lead contact upon request. The data are publicly available at GEO database. The accession numbers are listed in the key resources table.
• All original code is deposited on GitHub. The URL to the code is listed in key resources table.
• Any other information required will be provided by the lead author.

## Acknowledgments

This work was supported by NIH grants (P30 DK054759, RC2 DK124207, and NHLBI Federal Contract 75N92024C00008 to J.F.E.); 10.13039/100000897Cystic Fibrosis Foundation grant (ENGELH21XX0 to JFE) the Carver Chair in Molecular Medicine (to J.F.E.). Biorender was used for figure and graphical abstract schematics.

## Author contributions

Conceptualization, P.G.R. and J.F.E.; methodology, P.G.R., X.S., B.L., M.W., and Y.Z.; software, K.L.W. and N.C.; validation, P.G.R., G.G., F.Y., I.A.-E., and Y.Y.; formal analysis, P.G.R., Y.Y., G.G., F.Y., I.A.-E., P.W., G.L., S.C., R.R., A.E.S., and K.L.W.; investigation, P.G.R., G.G., F.Y., I.A.-E., and Y.Y.; formal analysis, P.G.R., Y.Y., G.G., F.Y., I.A.-E., P.W., G.L., S.C., R.R., A.E.S., and K.L.W.; resources, J.F.E., A.W.N., and L.S.; data curation, K.L.W.; writing – original draft, P.G.R. and J.F.E.; writing – review and editing, P.G.R., J.F.E., A.W.N., L.S., and K.L.W.; supervision, J.F.E., A.W.N., L.S., and A.U.; project administration, P.G.R., J.F.E., and A.W.N.; funding acquisition. J.F.E.

## Declaration of interests

The authors declare no competing interests.

## STAR★Methods

### Key resources table

**Table:** 

REAGENT or RESOURCE	SOURCE	IDENTIFIER
**Antibodies**		

Rabbit anti PDX1	Abcam	RRID: AB_777179
Mouse anti SOX9	Abcam	RRID: AB_2194156
Guinea Pig anti INS	Invitrogen	RRID: AB_794668
Rabbit anti CTNNB1	Abcam	RRID: AB_305407
Rabbit anti pSMAD5	Abcam	RRID: AB_10561456
Rabbit anti Axin2	Abcam	RRID: AB_2290204

**Bacterial and Virus Strains**		

pLenti6/V5-GW/LacZ	Thermo Fisher	Cat#K495510
One Shot Stbl3 Chemically Competent *E.coli*	Thermo Fisher	Cat#C737303

**Chemicals, Peptides, and Recombinant Proteins**		

Hoechst 33258	Molecular Probes	Cat#H3569
Accutase	Stem Cell Technologies	Cat#07920
Pneumacult Ex+	Stem Cell Technologies	Cat#05040
Pneumacult ALI	Stem Cell Technologies	Cat#05050
PTEN inhibitor VO-OHpic trihydrate	Sigma	Cat#8639
CHIR 99021 (GSK3b inhibitor/Wnt agonist)	Tocris	Cat#4423
Aqua-Mount	Thermo Scientific	Cat#13800
3-Isobutyl-1-methylxanthine (IBMX)	Sigma	Cat# 28822-58-4
Forskolin	Sigma	Cat# 66575-29-9

**Critical Commercial Assays**		

KAPA2G Robust PCR kit with dNTP 250 U	Roche Applied Science	Cat#07960743001
High-Capacity cDNA reverse transcription kit	Thermo Fisher	Cat#4368814
RNeasy plus mini kit	QIAGEN	Cat#74134
TaqMan Universal Master Mix	IDT	10007067
View RNA ISH kit	ThermoFisher Scientific	Cat#QVT0400
Illumina Tagment DNA Enzyme and Buffer Small Kit	Illumina	20034197
NEBNext High-Fidelity 2x PCR Master Mix	New England Biolabs	Cat#M0541S
Nextera DNA CD Indexes	Illumina	H503
AmpureXP Bead based Reagen	Beckman Coulter	A63881
MACS Dead Cell Removal Kit	Miltenyi Biotech	Cat#130-090-101
Lenti-X Concentrator	Takara	Cat#631232

**Experimental Models: Organisms/Strains**		

Ferret: CFTR^WT/WT^	Sun et al., 2010	N/A
Ferret: CFTR^G551D−KI/G551D−KI^	Yan et al., 2022	N/A
Ferret: CFTR^G551D/G551D^	Sun et al., 2019	N/A
Ferret: CFTR^G551D/KO^	Sun et al., 2019	N/A
Ferret: CFTR^KO/KO^	Sun et al., 2010	N/A

**Oligonucleotides**		

PDX1 primer 1: CCTCCCTTTGTCTTCCTTTTCC	This paper	N/A
PDX1 primer 2: ACCCTCGCAAGATGTTCTC	This paper	N/A
ACTB1 primer 1: TGAAGGTCTCGAACATGATCTG	This paper	N/A
ACTB1 primer 2: ACCACACCTTCTACAATGAGC	This paper	N/A
SOX9 primer 1: ACCTACACGGGCAGCTA	This paper	N/A
SOX9 primer 2: TGTAGTGGCTGGGACTCA	This paper	N/A
NKX6.1 primer 1: CAAACGAAATACTTGGCGGG	This paper	N/A
NKX6.1 primer 2: CGTGCTTCTTCCTCCACTTG	This paper	N/A
PAX6 primer 1: GAGTTATGACACCTACACCCC	This paper	N/A
PAX6 primer 2: ACATATCAGGTTCACTTCCAGG	This paper	N/A
CDH1 primer 1: GAGTGTGCCCCATTACCTAC	This paper	N/A
CDH1 primer 2: TCCCTTCATAGTCAAACACCAG	This paper	N/A
HNF6 primer 1: GAGGATGTGGAAGTGGCTG	This paper	N/A
HNF6 primer 2: ACATCTGTGAAGACCAACCTG	This paper	N/A

**Software and Algorithms**		

Analysis Code	This paper	https://github.com/kwells4/sussel_ferret_sc_220429
PyMiner	Tyler et al., 2019	N/A

**Deposited Data**		

Bulk RNA-seq of WT and CF PDE	This paper	GSE264019
Bulk ATACseq of WT and CF PDE	This paper	GSE264021
Single cell RNAseq of D2, D5, D7, D9 WT and CF PDE	This paper	GSE264022

### Experimental models and study participant details

#### Animal models

All procedures involving animals were performed in compliance with a protocol approved by the Institutional Animal Care and Use Committee of the University of Iowa and under institutional assurances including: AAALAC accreditation (#000833, since November 1994), USDA research facility registration (USDA No. 42-R-0004), and PHS Animal Welfare Assurance approval (D16–00009, A3021-01). Three ferret models of cystic fibrosis were used in these studies and express differing amounts of *CFTR*. The *CFTR* knockout ferret model (*CFTR*^KO^) was generated by somatic cell nuclear transfer using gene targeted fibroblasts.^111^ Homozygous *CFTR*^KO/KO^ ferrets (abbreviated as KO/KO) expresses no *CFTR* mRNA or protein. *CFTR*^KO/KO^ kits were reared as previously described.^129^ The *CFTR*^G551D^ ferret model was also generated by somatic cell nuclear transfer using gene targeted fibroblasts.^109^ This model is a hypomorph due to a selection cassette in the reverse orientation within the intron adjacent to the targeted exon. As a result, *CFTR*^G551D/G551D^ homozygous ferrets (abbreviated as G/G) expresses 50% *CFTR* mRNA and protein as compared to wildtype (WT) ferrets.^109^ *CFTR*^G551D/G551D^ kits were reared as previously described on the CFTR modulator (VX-770) from embryonic day 28.^109^ The *CFTR*^G551D−KI^ ferret model was generated by CRISPR/Cas9 targeting in zygotes.^110^ Unlike the *CFTR*^G551D/G551D^ ferret model, *CFTR*^G551D−KI/G551D−KI^ homozygous ferrets (abbreviated as GK/GK) have a ‘clean’ knock-in (KI) of the G551D mutation and express WT levels of *CFTR* mRNA. *CFTR*^G551D−KI/G551D−KI^ kits were reared as previously described on the CFTR modulator (VX-770) from embryonic day 28.^109^ CF ferrets harboring the G551D mutation are typically weaned off VX-770 at 1–2 months after birth, allowing for pancreatic remodeling to initiate in the absence of CFTR function.^2^ The equivalent pathophysiologic state for a 2-months-old CFTR knockout ferret would be a 3-months-old *CFTR*^G551D−KI/G551D−KI^ ferret harboring removed from VX-770 at 1 month of age.^2^ We used animals with a CF age around 1.5-month, which is the peak of inflammation and fibrosis. WT ferrets of sable coat color were obtained from Marshall Farms (North Rose, NY). Various allelic *CFTR* mutation combinations were generated through cross-breeding of the CF ferret lines. Offspring from this cross-breeding gave rise to ferrets with various levels of CFTR expression (Table 1).

**Table 1:** CF ferret line names and abbreviation associated with various levels of CFTR expression

Formal line name	Abbreviated name	Level of WT CFTR expression
CFTR^WT/WT^	WT	100%
CFTR^G551D−KI/G551D−KI^	GKGK	100%
CFTR^G551D/G551D^	GG	50%
CFTR^G551D/KO^	GKO	25%
CFTR^KO/KO^	CFTR-KO, CFKO, or KO	0%

### Pancreatic ductal cell harvest, propagation, and differentiation at an ALI

Pancreata were removed from 1 to 3 day old newborn WT and CF ferrets and digested in 5 mg/mL collagenase for 20 min at 37^°^C. The digested pancreas was incubated overnight in PneumaCult-Ex Plus medium (STEMCELL Technologies, MA, USA) on 804G-coated culture dishes overnight at 37^°^C in a 5% CO_2_ incubator. The 804G coating procedure is as previously described for airway basal cells.^130^ Ductal structures that adhered to the plate were aspirated on the following day and cultured on fresh 804G-coated culture dishes in PneumaCult-Ex Plus medium until near confluence. These cells were then passaged by incubating with Accutase (STEMCELL Technologies, MA, USA) for 5 min at 37^°^C. The cells were passaged continuously for 10 passages to eliminate contaminating cells and obtain a morphologically homogeneous population of duct cells. Passage-10 cells were harvested using Accutase and transferred to 804G-coated transwell inserts (Corning, NY, USA) at a density of 100,000 cells per well. Following seeding, transwells were cultured in PneumaCult-Ex Plus medium on both apical and basolateral chambers for 3 days. The medium in both the apical and basolateral chamber was then switched to PneumaCult-ALI (STEMCELL Technologies, MA, USA) for 1 day. Air liquid interface (ALI) was established the following day by aspirating the medium from the apical chamber. Cultures were maintained at an ALI for 2 weeks before use in experiments at which time transepithelial resistance should be greater than 1000 Ω. Cells in culture were tested for mycoplasma contamination regularly (once every two months).

### Method details

#### Immunofluorescence staining of differentiated epithelial cultures

Differentiated cultures of WT and CF pancreatic ductal epithelium (PDE) were fixed in 4% paraformaldehyde (PFA) for 20 min at room temperature (RT). After 3 washes in 1X PBS for 5 min, the cells were incubated in blocking solution containing 10% donkey serum in PBS for 1 h at RT. Primary antibodies were diluted and applied overnight at 4^°^C at the following dilutions (CTNNB1, 1:100; PSMAD5, 1:100; INS, 1:500). Slides were washed in 1X PBS thrice for 15 min each and incubated with secondary antibodies (anti-rabbit Alexa 594, A21207, Invitrogen, USA; anti-mouse Alexa 594, 715-585-151, Jackson ImmunoResearch, USA) at a 1:1000 dilution for 1 h at RT. Slides were mounted using Aquamount (Thermo Scientific,MA,USA) containing Hoescht diluted at 1:2000 and imaged on a Zeiss 880.

#### Immunofluorescence staining of pancreatic sections

Paraffin sections from newborn or 1- to 2-month old WT and CF ferrets were deparaffinized and dehydrated by sequential 5 min incubation in xylene (3 times) and 100% ethanol (2 times). The sections were rehydrated by sequential 5 min incubations in 90%, 70%, 50%, 30% ethanol followed by water (2 times). Sections in citrate-based antigen retrieval solution were incubated in a water bath at 95°C for 1 h. The slides were placed at RT for 40 min and then incubated in blocking solution (1X PBS with 10% donkey serum) for 1 h at RT. Primary antibodies were applied overnight at 4^°^C at the following dilutions (PDX1, 1:100; SOX9, 1:100; AXIN2 1:100; INS, 1; 100). The slides were washed for 15 min in 1X PBS thrice. The secondary antibodies were diluted to 1:2000 and applied for 1 h at RT. The slides were washed for 5 min in 1X PBS thrice and mounted using Aquamont containing Hoescht diluted at 1:2000 and imaged on a Zeiss 880.

#### RNA fluorescent *in situ* hybridization (FISH) on pancreatic sections

FISH was used to visualize mRNA expression using ViewRNA ISH kits (Thermo Fisher, MA, USA) on paraffin embedded sections of WT and *CFTR*-KO ferret pancreata. Prior to deparaffinization the pancreatic sections were baked at 60^°^C for 1 h. During deparaffinization sections were submerged in xylene three times for 5 min, dehydrated in 100% ethanol twice for 5 min, followed by air-drying. Tissue was permeabilized by incubating the sections in permeabilizing solution (Thermo Fisher, MA, USA) for 10 min at 90-95^°^C. The sections were then incubated in protease solution (Thermo Fisher, MA, USA) at a 1:100 dilution for 10 min at 40^°^C. Oligonucleotide probes (Thermo Fisher, MA, USA) for ferret transcripts (*INS, RNASE1, AMY2B, HNF6, WNT5A, TGF-β*) were diluted to 1:40 (except *INS* at 1:200) with target probe diluent buffer and incubated with the sections for 5 h at 40^°^C, followed by three rigorous washes using the wash buffer provided in the kit for 15 min each. Sections were incubated in pre-amplifying buffer at 40^°^C for 25 min, followed by three rigorous washes for 15 min each. Sections were then incubated with amplifying solutions at 40^°^C for 15 min, followed by three washes lasting 15 min each. Sections were then incubated in labeling probe buffer mixture diluted to 1:1000 at 40^°^C for 30 min, washed, and developed using Blue or Red substrate bound to the alkaline phosphatase enzyme. Sections were mounted using Hoescht containing (1:2000) Aquamount (Thermo Scientific, MA,USA) and stored at 4^°^C until they were imaged on a Zeiss 880 Confocal.

#### Short-circuit current (isc) measurements on PDE cultures

CFTR dependent chloride current measurements on WT and CF PDE cultures were performed using Ussing chambers as previously described.^109^ Changes in Isc were assessed after the sequential addition of the following channel agonist and antagonists to the apical chamber: 1) Amiloride (100 μM) to inhibit ENaC, 2) 4,4′-Diisothiocyano-2,2′-stilbenedisulfonic acid (DIDS,100 μM) to inhibit non-CFTR Cl^−^ channels, 4) 8-Methoxymethyl-3-isobutyl-1-methylxanthine (IBMX, 100 μM) and Forskolin (10 μM) to stimulate CFTR, and 4) GlyH101 (3 μM) to inhibit CFTR. Amiloride and DIDS inhibit the sodium channels and non-CFTR chloride channels, respectively. IBMX and Forskolin both stimulate increases in intracellular cAMP and activates the CFTR channel. Isc were recorded using Acquire and Analyze software (Physiologic Instruments, CA, USA).

#### Quantitative qRT-PCR on proliferating pancreatic ductal cells (PDC) and differentiated PDE cultures

RNA was harvested from PDCs and PDEs (2–4 transwells combined per donor) using Qiagen RNEasy Plus Kits (Qiagen, MD, USA). Equal amounts of RNA were used to generate cDNA using the High-capacity cDNA Reverse Transcription kit (Thermo Fisher, MA, USA). Taqman probes were designed against the ferret transcripts for genes of interest (*PDX1*, *SOX9*, *PAX6*, *NKX6.1*, *HNF6*, *KRT7*, *CDH1, ACTB*) using the IDT tools website (IDT, IA, USA). The Taqman Real-time PCR Master Mix (IDT, IA, USA) was mixed with 2 μL of cDNA. RT-qPCR was performed on the BioRAD thermocycler (BioRAD, CA, USA). The PCR cycling conditions included a denaturation step at 95^°^C for 3 min followed by 41 cycles of 95^°^C for 15 s and 60^°^C for 45 s. Blanks and negative control samples excluding the reverse transcriptase were used in every experiment. *ACTB* was used as the internal control housekeeping gene for normalization.

#### PTEN and GSK3β inhibitory studies on actively polarizing WT PDE cultures

Actively polarizing WT PDEs were used to test whether inhibition of PTEN or GSK3β pathways led to a *CFTR*-KO phenotype. Approximately 100,000 PDCs were plated on 804G coated *trans*-wells (Corning, NY, USA). Cells were incubated for three days in PneumaCult-Ex Plus in both the apical and basolateral chambers. The media was then replaced with PneumaCult-ALI in both apical and basolateral chambers for overnight incubation. The next day the apical media was then aspirated and PTENi or GSK3βi was added to the basolateral media at 10 μM concentration; Donor-matched controls received vehicle. The cells were sustained for 2 weeks in the presence of the inhibitors. Subsequently, 2–4 *trans*-wells were combined to harvest RNA using the RNEasy Plus Kit (Qiagen, MD, USA). Three samples (combining 2–4 *trans*-wells per sample) per donor per condition were used for subsequent assays.

#### CFTR complementation studies in *CFTR*-KO PDEs

Lentivirus PGK-hCFTR-dTomato was previously described as pLV-dt/CFTR-Ø.^131^ It contains a phosphoglycerate kinase 1 (PGK) promoter driving CFTR and CMV beta-actin promoter driven dTomato cassettes. PGK-dTomato (control) virus was generated from pLV-dt/CFTR-Ø by deleting the CFTR gene. Proviral plasmids were amplified using Stbl3 competent E.Coli (Thermofisher, MA, USA). Lentivirus was generated as previously described.^131^ PDCs at passage 8–10 from *CFTR-*KO donors were transduced with Lenti-PGK-hCFTR-dTomato or Lenti-PGK-dTomato at an MOI of 100 particles/cell. Cells were passaged for 7 days and then dTomato positive cells were isolated by FACS and expanded for experiments. PDCs were then plated onto tranwells and polarized for 2 weeks at an ALI in PneumaCult-ALI media. RNA was then generated from 2 to 4 transwells combined.

#### Bulk RNA-seq sample preparation from differentiated PDE cultures

WT and CF PDE cultures differentiated from 3 donors of each genotype were polarized for 2 weeks at an ALI. Triplicate samples per donor were prepared by combining two transwells per replicate to extract RNA from the cells using RNeasy Plus Mini Kit (Qiagen, MA, USA). Genomic DNA was eliminated using gDNA columns (Qiagen, MA, USA). Extracted RNA was analyzed for integrity by assessing their RNA integrity number. Samples with RIN >9 were used to prepare indexed libraries using Trueseq mRNA stranded preparation. Barcoded samples were pooled in equimolar rations and sequenced for 75 bp paired end reads using a HiSeq4000 (Illumina) in the University of Iowa Genomics Division.

#### ATAC-seq sample preparation from differentiated PDE cultures

WT and CF PDE cultures differentiated from 3 donors of each genotype were polarized for 2 weeks at an ALI. Samples were processed for ATAC-seq according to previously published protocols.^132^^,^^133^ Cells were dissociated using Accutase and ∼50,000 cells from each PDE culture were lysed in ice-cold lysis buffer (10 mM Tris-HCl, pH 7.4, 10 mM NaCl, 3 mM MgCl2, 0.1% NP-40: Sigma). Transposition was performed using 25 μL tagmentation reaction mix from Tagment DNA kit (Illumina, CA, USA). Tagged DNA was amplified indexed, using the NEBNext High-Fidelity 2x PCR Master Mix (New England Biolabs, MA, USA) and with Nextera DNA CD Indexes (Illumina, CA, USA), using the following settings: 72°C for 5 min; 98°C for 30 s; 12 cycles of 98°C for 10 s, 63°C for 30 s, and 72°C for 1 min. The indexed library was purified with 1.8 times the volume of Ampure XP beads (Beckman Coulter, CA, USA). Library quality was assessed using a BioAnalyzer 2100 High Sensitivity DNA Chip (Agilent Technologies, MA, USA). All DNA libraries that exhibited the correct nucleosome pattern were pooled and processed for 150bp paired-end sequencing using a HiSeq4000 (Illumina) in the University of Iowa Genomics Division.

#### Single cell RNA-seq sample preparation

WT and CF ferret PDE cultures grown at ALI for 2, 5, 7, and 9 days were dissociated using Accutase followed by DNase treatment. Cells were filtered through a 20 μM strainer and pelleted in 0.04% BSA PBS at 500 g for 10 min. Nonviable dead cells were removed by using MACS Dead Cell Removal Kit following 10X Genomics recommendations (Document CG00039). Single cells were counted on a Thermo countess cell counter and 0.04% BSA/PBS was added to achieve a targeted concentration of 1000 cells/μl. Sequencing libraries were generated by following 10X Genomics recommendations (Document CG000315). Briefly, single cells and reverse transcription master mix were partitioned into Gel Beads in partitioning oil in the 10X Chromium controller. After reverse transcription, cDNA libraries were amplified and fragmentated, followed by adaptor ligation and sample index PCR reaction. Libraries were sequenced on NovaSeq 6000 platform by the University of Iowa Genomics Division.

### Quantification and statistical analysis

#### Image analysis

Image quantification was performed using FIJI. Maximum intensity projection of Z-stacks with at least 15 sections were generated. Regions of interest were manually drawn around ductal structures for quantification of signal intensity and area. Fluorescent signals overlapping with DAPI stain was quantified for nuclear localization. At least 3 donors per condition were used for quantification and statistical analysis. Statistical analysis of the measurements was done using Graphpad PRISM.

#### Analysis of WT and CF PDE bulk RNA-seq

FASTQ reads obtained after sequencing were mapped to the ferret reference genome using RSEM. The normalized transcripts per million counts for each gene was generated. Differentially expressed genes (DEG) with BH corrected *p*-value <0.05 were obtained using PyMiner.^17^ The list of DEGs were used to perform subsequent pathway analysis and upstream regulator analysis on Ingenuity Pathway Analysis (Qiagen Bioinformatic).

#### ATAC-seq peak calling and differential analysis

ATAC-seq was performed using 150 bp paired-end sequencing reads. Raw FASTQ reads were trimmed using Trimmomatic on Galaxy tools and aligned to ferret genome assembly (MusPutFur1.0) using Bowtie 2 on Galaxy tools with default parameters. DeepTools version 3.3.0 was used to check the reproducibility of the biological replicates and generate bigWig coverage files for visualization. MACS2 was used to call peaks. DiffBind version 2.10 was used to identify differential peaks between WT and CF PDEs with log2 fold-change threshold of >1 and a false discovery rate (FDR) < 0.05, indicating differential accessibility. Peaks were assigned to genes using BED intersect, to identify differentially accessible genes. IGV was used to visualize differentially accessible regions of the genome.

#### Single cell RNA-seq analysis of WT and CF PDE

##### Pre-processing

Sequences from scRNA-seq were processed using 10x Genomics Cellranger v 5.0.1 software^134^ using the ferret ASM1176430v1.1 genome and gtf file. A recently described improved annotation of the ferret transcriptome was used which achieves a median read assignment in scRNA-seq studies of ∼71%.^127^ The genome and gtf file were made into a cellranger reference using the `mkref’ command.

Raw data generated by Cellranger were then read into R v4.1.2 using the Seurat^135^ v4.1.1 R package with at least 200 genes per cell and at least 3 cells. Cells were further filtered based on the number of genes per cell and the percent of mitochondrial reads per cell (maximum 20%). The data were normalized by using `NormalizeData’. For each sample, variable genes were found by using `FindVariableFeatures’ and data was scaled using `ScaleData’.

Doublets were removed using `DoubletFinder’^136^ using the default values except for pK, nExp, and PCs. The pK was identified using the pK associated with the maximum BCmetric value after running `find.pK’ from doublet finder. All samples used PCs 1:20. After doublet removal, 4499 (CFKO_D2), 2750 (CFKO_D5), 1607 (CFKO_D7), 2489 (CFKO_D9), 6224 (WT_D2), 5326 (WT_D5), 4634 (WT_D7), 5416 (WT_D9) cells were used for downstream analysis.

The 8 samples were merged using the `merge’ function from Seurat. The merged data was then normalized and scaled using `NormalizeData’, `FindVariableFeatures’, and `ScaleData’ as described above. Dimensionality reduction and clustering were performed using `RunPCA’, `FindNeighbors’, `FindClusters’, and `RunUMAP’. `RunPCA’ was run using the default values except all variable features were used for the features argument. `FindNeighbors’ was run with default parameters except for the dims argument (dims = 1:30). `FindClusters’ was run with default parameters except for the resolution argument (resolution = 0.6). `RunUMAP’ was run with default parameters except for the dims and metric arguments (dims = 1:30, metric = “correlation”). Because some clusters had good representation of all development days and we wanted to analyze the WT and KO separately, no batch correction was performed.

##### Cell type identification

We made a first pass at naming clusters on the merged WT and merged CFKO samples separately using a mix of published single cell studies of mouse and human pancreas^62^^,^^63^^,^^64^^,^^65^^,^^66^^,^^137^ as a reference and determining cluster identity using `clustifyr’.^61^ For each reference, we used the top 2000 variable genes and found either the mouse or human ortholog. These orthologs were used as the `query_genes’ and the clusters identified by `FindClusters’ as the `cluster_col’. The top correlated cell type from any reference to each cluster was used as the first determination of cell type.

##### Cluster markers and pathways

To identify markers of each cluster, `FindAllMarkers’ was run on each merged dataset using default settings except we set only.pos to TRUE. Genes were called differentially expressed if the adjusted *p*-value was less than 0.05 and the log fold change was greater than 0.5. To generate a heatmap of gene sets, the average expression of all genes within each cluster was determined using `AverageExpression’ from Seurat. Next, the average expression matrix was subset to only the genes in the gene set of interest. Finally, a *Z* score was computed, and the plot was generated with `pheatmap’ (https://cran.r-project.org/web/packages/pheatmap/index.html).

##### Trajectory analysis

To perform trajectory analysis, we used `slingshot’. We identified starting clusters based on the cluster containing the most D2 cells for each WT and CFKO. Slingshot was run on the first 30 PCs of each dataset. We then used `tradeseq’ using the pseudotime scores generated by slingshot to identify genes that correlated with pseudotime. We used the function `evaluateK’ to determine the number of knots (5 for the CFKO, 8 for WT) and the function `fitGAM’ to identify correlated genes. To visualize pseudotime on the UMAP, we used the `EmbedCurves’ function to map our pseudotime data onto the UMAP dimensionality reduction.

##### Figures

Most figures were created using the `scAnalysisR’ package available on github (https://github.com/CUAnschutzBDC/scAnalysisR). All scripts to replicate this analysis are available on github (https://github.com/kwells4/sussel_ferret_sc_220429).

## Supplemental information

Document S1. Figures S1–S6

Table S1. Bulk RNA-seq of WT and CF primary PDEs, related to Figure 2 (A) Bulk RNA sequencing analysis results of WT (*n* = 3) and CF (*n* = 3) PDEs. (B) Upstream Regulator analysis showing transcriptional regulators of DEGs in CF PDEs using IPA. (C) Upstream regulator analysis results for DEG in CF PDEs.

Table S2. GO Term analysis on differentially expressed genes in CFTR-KO PDE, related to Figure S2 (A) Results from GO term analysis on DEG in CFTR-KO PDE using Panther. (B) Gene list associated with cell differentiation GO term. (C) Gene set associated with cell migration GO term. (D) Gene set for mesenchyme development GO term. (E) Gene set for response to TGF-β stimulus GO term. (F) Gene set for response to WNT signaling is listed. (G) Gene set for endocrine pancreas development GO term. (H) Gene set for histone modification GO term.

Table S3. Bulk ATAC-seq of WT and CF primary PDEs, related to Figure 3 (A) The differentially open regions (DORs) associated with coding regions in the chromatin of CF PDEs relative to WT PDEs are shown in the table. (B) Analysis of motif enrichment in the CF DORs.

Table S4. Differentially expressed genes (DEGs) in subpopulations derived from WT and CF PDE, related to Figure 4 (A) DEG in acinar cells derived from WT and CF PDE. (B) DEG in ductal cells found in WT and CF PDE. (C) DEG in proliferating acinar cells derived from WT and CF PDE. (D) DEG in proliferating ductal cells derived from WT and CF PDE. (E) DEG in centroacinar progenitor cells derived from WT and CF PDE. (F) DEG in centroacinar cells derived from WT and CF PDE.

Table S5. DEGs at the D2, D5, D7, and D9 during the differentiation of WT and CF PDEs, related to Figure 4 (A) DEGs in CF PDEs relative to WT PDEs at D2 of differentiation. (B) DEGs in CF PDEs relative to WT PDEs at D5 of differentiation. (C) The DEGs in CF PDEs relative to WT PDEs at D7 of differentiation. (D) The DEGs in CF PDEs relative to WT PDEs at D9 of differentiation.

Table S6. Variability in DEG expression across all time points of differentiation (A) The variability in expression of D2 DEGs. (B) The variability in expression of D5 DEGs. (C) The variability in expression of D7 DEGs. (D) The variability in expression of D9 DEGs. (E) The variability in expression of D9 CAC progenitor DEGs. (F) The variability in expression of D9 CAC DEGs.

Table S7. Upstream regulator analysis and pathway analysis of DEGs during WT and CF PDE differentiation (A) Upstream regulator analysis for DEGs at D2 of differentiation using IPA. (B) Upstream regulator analysis for DEGs at D5 of differentiation using IPA. (C) Upstream regulator analysis for DEGs at D7 of differentiation using IPA. (D) Upstream regulator analysis for DEGs at D9 of differentiation using IPA. (E) Pathway analysis for CF PDE DEGs at D2 of differentiation. (F) Pathway analysis for CF PDE DEG at D5 of differentiation. (G) Pathway analysis for CF PDE DEGs at D7 of differentiation. (H) Pathway analysis for CF PDE DEGs at D9 of differentiation.

Table S8. DEGs in ductal subpopulations, related to Figure S6 (A) The DEGs in WT ductal 1 subpopulation. (B) The DEGs in WT ductal 2 subpopulation. (C) The DEGs in WT ductal 3 subpopulation. (D) The DEGs in CF ductal 1 subpopulation. (E) The DEGs in CF ductal 2 subpopulation are shown. (F) The DEGs in CF ductal 3 subpopulation are shown. (G) The *Z* scores of DEGs in ductal subpopulations identified in WT and CF PDEs. (H) The DEGs in ductal cells of WT and CF PDEs, and their *Z* scores for expression across all time points of differentiation.

Table S9. Upstream regulator analysis of DEGs in D9 CACs and CAC progenitors, related to Figure S5 (A) The predicted upstream regulatory signaling pathways active in CF CACs and relative to WT CACs. (B) The predicted upstream regulatory signaling pathways active in CF progenitor-like cells relative to WT progenitor-like cells. (C) The *Z* score enrichment values for AKT signaling genes in WT and CF PDE cells at D9 differentiation. (D) The *Z* score enrichment values for PTEN signaling genes in WT and CF PDE cells at D9 differentiation. (E) The *Z* score enrichment values for WNT signaling genes in WT and CF PDE cells at D9 differentiation.

Table S10. Bulk RNA-seq of pancreatic ductal cells derived from WT and CF PDE, related to Figure 2
