# Supplementary material for: CFTR represses a PDX1 axis to govern pancreatic ductal cell fate
Source: iScience. 2024 Nov 15;27(12):111393. doi: 10.1016/j.isci.2024.111393 (PMC11647141; doi:10.1016/j.isci.2024.111393)
Supplement: Document S1. Figures S1–S6 [file mmc1.pdf]

iScience, Volume *27*

Supplemental information

CFTR represses a PDX1 axis

to govern pancreatic ductal cell fate

Pavana G. Rotti, Yaling Yi, Grace Gasser, Feng Yuan, Xingshen Sun, Idil Apak-Evans, Peipei Wu, Guangming Liu, Soon Choi, Rosie Reeves, Attilina E. Scioneaux, Yulong Zhang, Michael Winter, Bo Liang, Nathan Cunicelli, Aliye Uc, Andrew W. Norris, Lori Sussel, Kristen L. Wells, and John F. Engelhardt

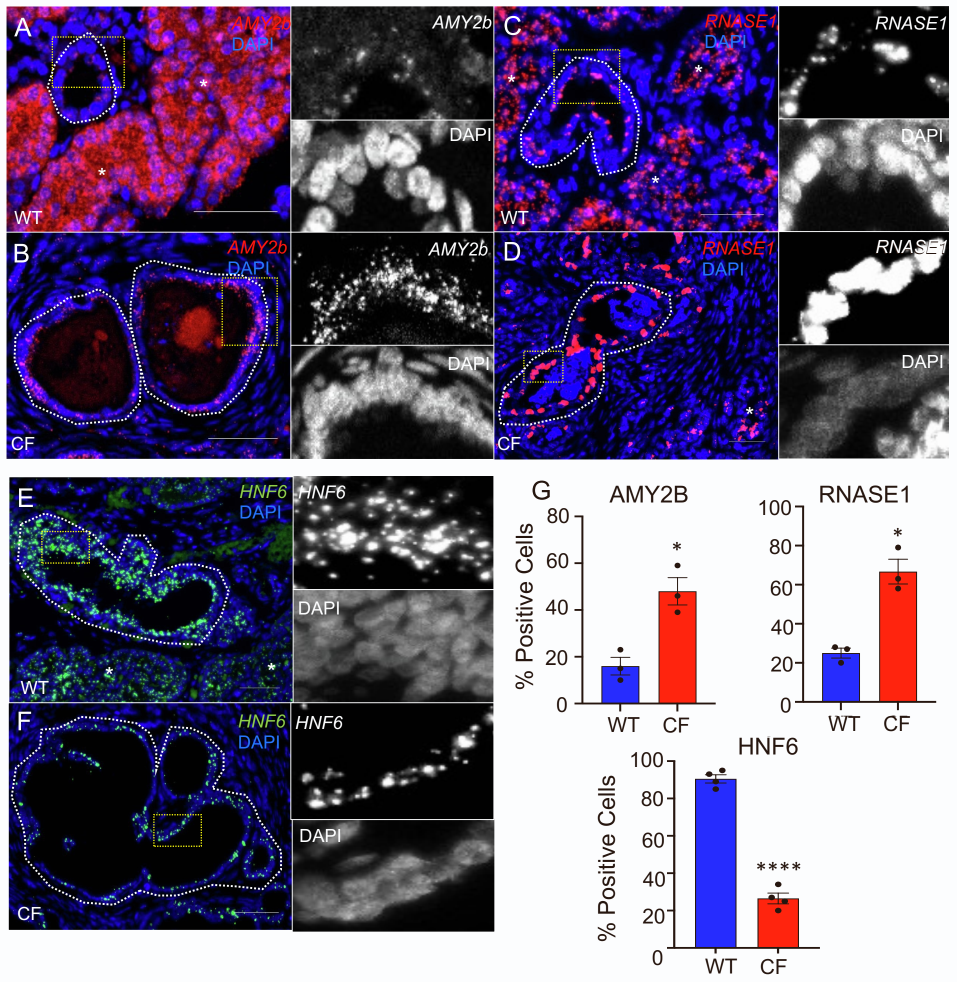

Figure S1. CF pancreatic ductal epithelia have enhanced expression of acinar markers genes (Related to Figure 1 and 2). (A,B) Fluorescent in situ hybridization (FISH) for acinar markers (A,B) *AMY2B* and (C,D) *RNASE1* mRNA expression in (A,C) WT and (B,D) CF 2-month old ferret pancreas. (E,F) FISH for ductal marker *HNF6* mRNA expression in (E) WT and (F) CF 2-month old pancreas. Ducts are identified by the presence of a lumen and are indicated by bound dotted lines. Scale bars are 50 μm. Magnified single channel images of the boxed regions are shown as insets. Images were obtained on confocal microscope Zeiss 880 at 20X magnification and processed for maximum intensity projection. (G) Quantification of % positive *AMY2B, RNASE1 and HNF6* expressing cells in N=3 WT and CF donors. Error bars show standard error mean. Significance was calculated using t-test with Welsch's correction (*$p<0.05$, ****$p<0.0001$)

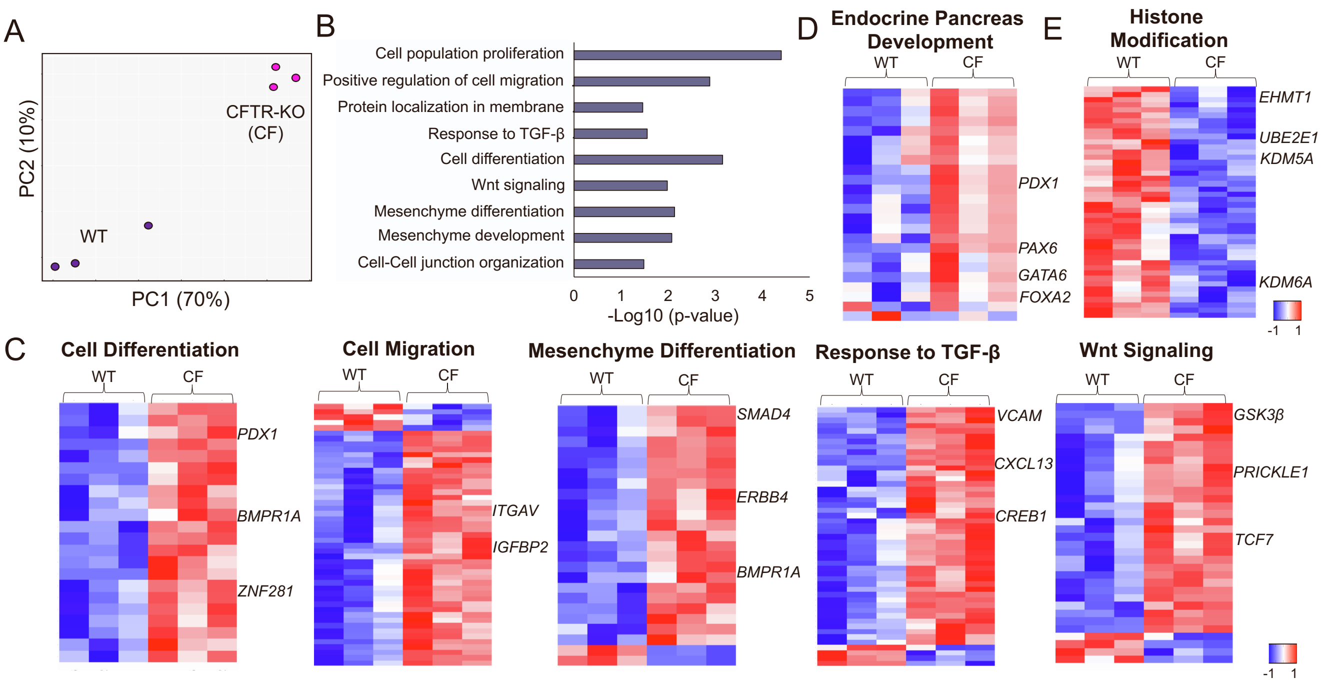

Figure S2. Bulk transcriptome analysis of WT and CF PDE cultures (Related to Figure 2). (A) Principal component analysis (PC) of genes expressed in WT and *CFTR*-KO (CF) PDE cultures. (B) List of GO-Term annotations for the differentially expressed genes (DEGs). (C) Heatmaps of enrichment values (Z-scores) for DEG genes associated with GO Terms related to cell differentiation, cell migration, mesenchyme differentiation, response to TGF-β , and Wnt signaling. (D) Heatmap of enrichment values (Z-scores) of genes associated with pancreatic endocrine differentiation with motifs enriched in the differentially open regions of the genome in CF PDEs by ATAC-seq. (E) Heatmap of enrichment values (Z-scores) for DEG genes associated with GO Term Histone Modification.

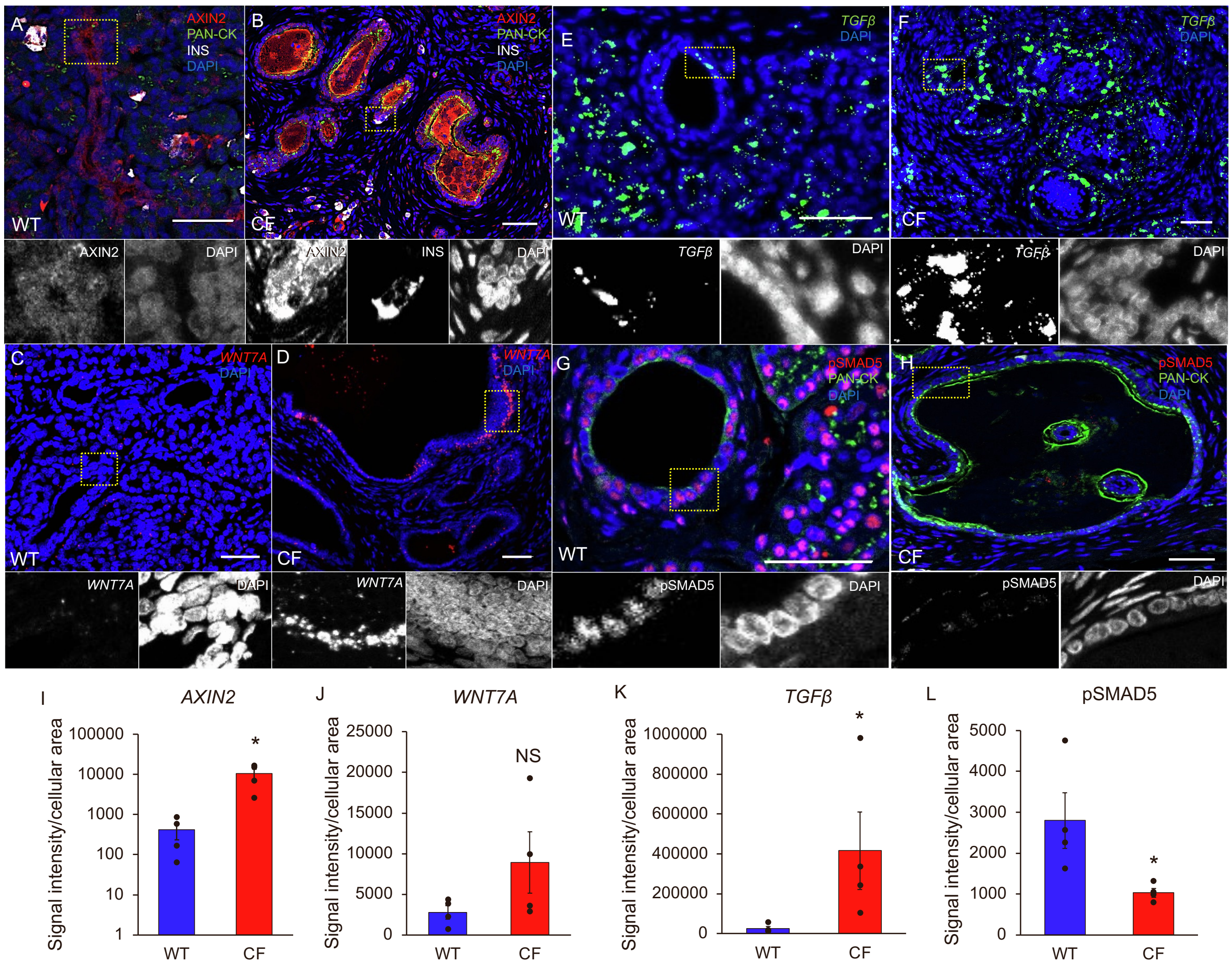

Figure S3. Characterization of candidate signaling pathways altered in CF pancreas (Related to Figure 2). (A,B) Localization of WNT signaling target (AXIN2), INS, and pan-CK in 2-month old (A) WT and (B) CF ferret pancreas. (C-F) FISH localization of (C,D) *WNT7A* and (E,F) *TGFB* mRNA in ducts of 2-month old (C,E) WT and (D,F) CF pancreas. (G,H) Nuclear localization of pSMAD5 and pan-CK in ducts of 2-month old (G) WT and (H) CF ferret pancreas. Insets are magnified single channel images of the boxed regions. Insets in D and E are rotated 90 degrees clockwise. Images were obtained on confocal microscope Zeiss 880 at 20X magnification and processed for maximum intensity projection. Scale bars are 50 μm. (I-L) Quantification of (I) AXIN2, (J) *WNT7A*, (K) *TGFB* and (L) pSMAD5 staining. Ducts were identified by the presence of a lumen were manually cropped out for the quantification. Average intensity of AXIN2, *WNT7A*, and *TGFB* was normalized to the number of cells. Only pSMAD5 signal that colocalized with DAPI signal was quantified. Mean intensity was normalized to the number of cells (DAPI). Data shown is the average +/- SEM (n=3 donors per genotype). At least 3 ducts were analyzed per donor per genotype. Significance was calculated using non-parametric Mann-Whitney t-test (*$p<0.05$). Note: luminal secretions and cellular debris in CF duct had significant background staining with the AXIN2 antibody.

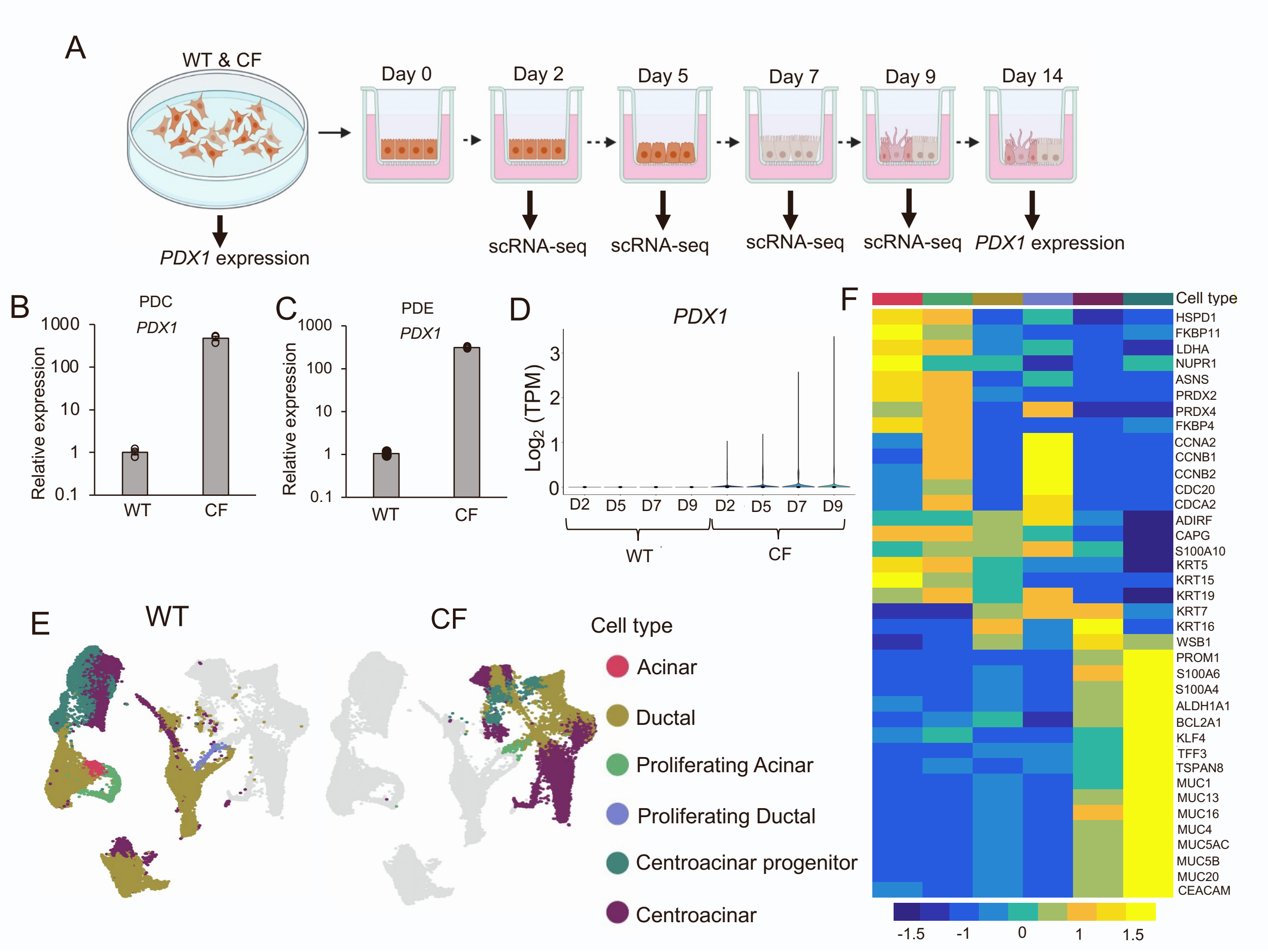

Figure S4. scRNA-seq of actively differentiating WT and CF PDE cultures (Related to Figure 4). (A) Schematic of samples preparation for scRNA-seq. Cultures of WT and CF PDCs were differentiated at air-liquid interface for 14 days. Samples from day 2, 5, 7 and 9 were processed for scRNA-seq. *PDX1* expression was also evaluated by RT-qPCR before differentiation before differentiation and on day-14 of the differentiation. (B,C) *PDX1* mRNA expression in WT and CF PDCs (B) prior to differentiation and (C) at day-14 of differentiation (n=3 donors per genotype). Data shows mean expression level +/- SEM. (D) Normalized counts of *PDX1* transcripts by scRNA-seq. Higher *PDX1* expression was observed in a small subset of CF PDE cells. (E) UMAPs of single sequenced cells from WT and CF PDEs (all timepoints). The mapped cell types are color-coded on the right based on the top 2000 cell marker genes from combined human and mouse pancreatic datasets. (F) Heatmap showing the expression of cell type marker genes in all mapped populations.

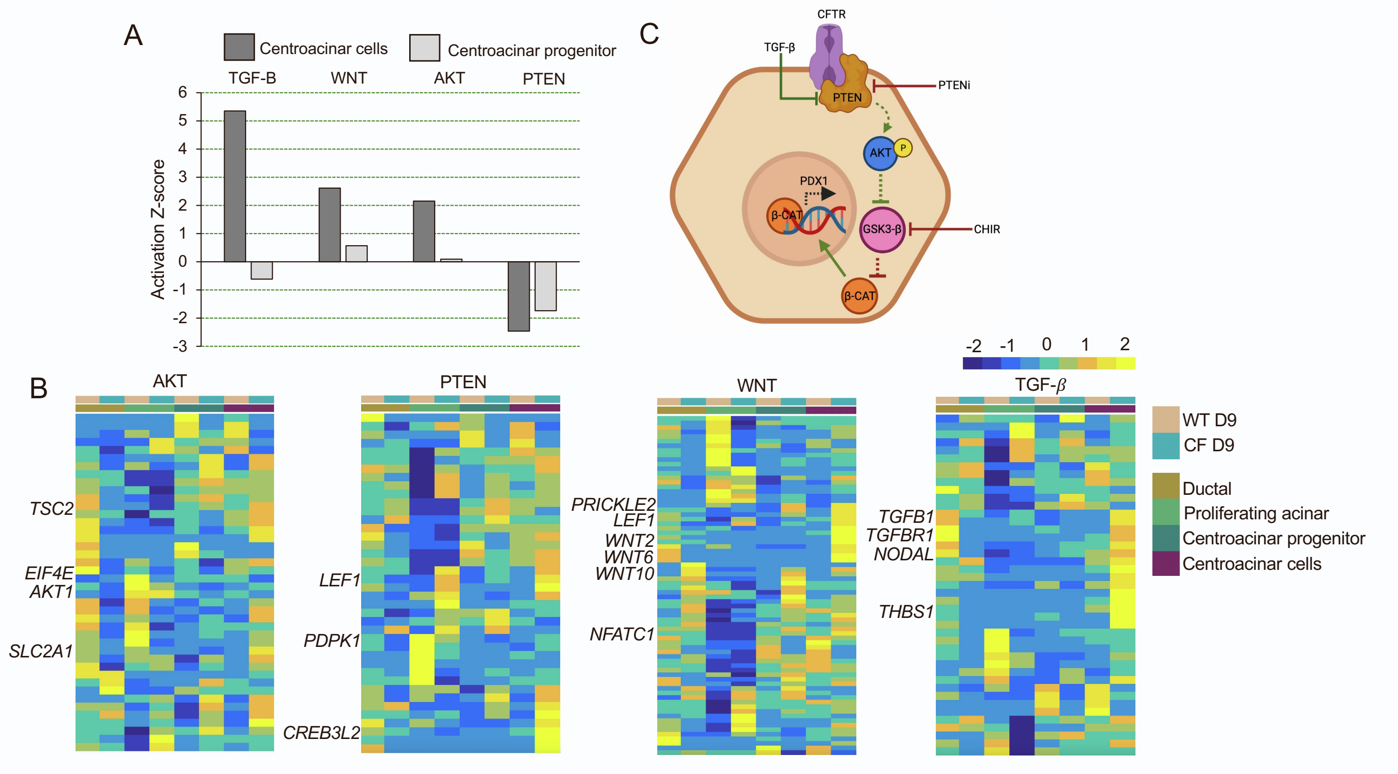

Figure S5. Altered signaling pathway genes dysregulated in CF centroacinar cell lineages (Related to Figure 4 and 5). (A) Activation Z-scores of putative upstream regulators for DEGs in centroacinar cells and centroacinar progenitor cells found by scRNA-seq WT and CF differentiating PDE cultures. Activation of pathways indicated by Z-score > 2 and inhibition by Z-score < -2. Activation of TGFB, WNT and AKT pathways were observed only in centroacinar cells. Similarly, inhibition of PTEN was observed only in centroacinar cells. (B) Expression of AKT, PTEN, TGFB and WNT pathway associated genes found in the KEGG database for WT and CF day 9 (D9) PDE cultures. Genotype and cell phenotype annotations are colored coded on the top of each heatmap. Genes relevant to disease progression in CFRD are highlighted. (C) Proposed model of the signaling mechanism responsible for activation of *PDX1* expression in CFTR-KO PDEs. Elevated TGFB expression in CFTR-KO pancreatic ductal epithelia acts to inhibit PTEN [138]. The lack of CFTR protein at the membrane further leads to inhibition of PTEN activity and thus pathway inactivation of GSK3β.

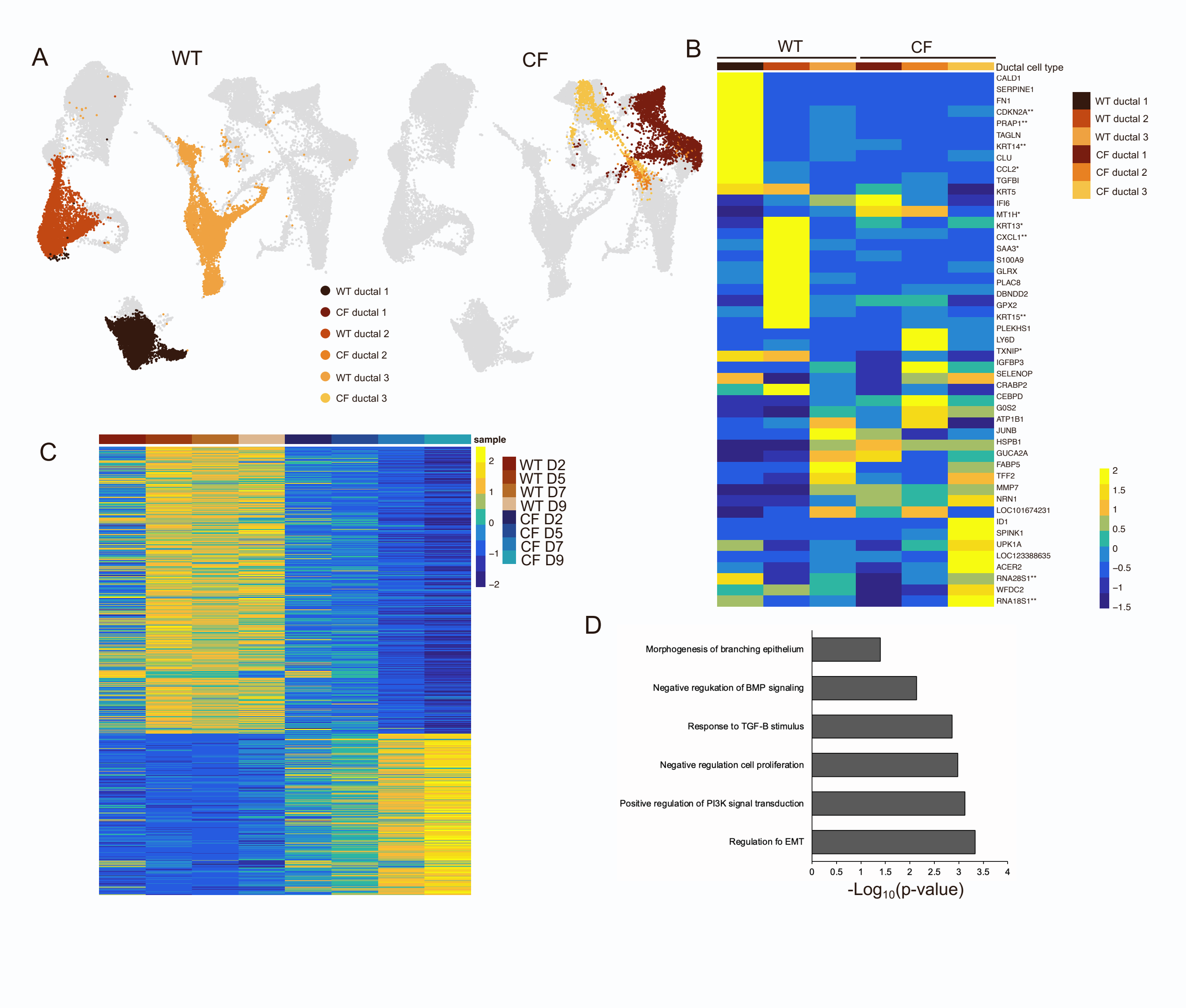

Figure S6. Characterization of ductal subpopulations in WT and CF PDEs (Related to Figure 4). (A) UMAPs showing ductal subpopulations identified in WT and CF PDE at various stages of differentiation. (B) Heatmap shows DEGs in shown ductal subpopulations at various stages of differentiation. (C) Heatmap indicates the DEGs in all WT and CF PDE ductal cells. (D) GO-term analysis of DEGs from WT and CF PDE ductal cells.
